# Leeno: Type 1 diabetes management training environment using smart algorithms

**DOI:** 10.1371/journal.pone.0274534

**Published:** 2022-09-15

**Authors:** Mohamed Raef Smaoui, Ahmad Lafi

**Affiliations:** Department of Computer Science, Faculty of Science, Kuwait University, Kuwait City, Kuwait; Universidad de Granada, SPAIN

## Abstract

A growing number of Type-1 Diabetes (T1D) patients globally use insulin pump technologies to monitor and manage their glucose levels. Although recent advances in closed-loop systems promise automated pump control in the near future, most patients worldwide still use open-loop continuous subcutaneous insulin infusion (CSII) devices which require close monitoring and continuous regulation. Apart from specialized diabetes units, hospital physicians and nurses generally lack necessary training to support the growing number of patients on insulin pumps. Most hospital staff and providers worldwide have never seen or operated an insulin pump device. T1D patients at nurseries, schools, in hospital emergency rooms, surgery theatres, and in-patient units all require close monitoring and active management. The lack of knowledge and necessary training to support T1D patients on pumps puts them at life-threatening risks. In this work, we develop a training simulation software for hospitals to educate and train their physicians and nurses on how to effectively operate a T1D pump and reduce hypoglycemia events. The software includes clinically validated T1D virtual patients that users can monitor and adjust their pump settings to improve glycemic outcomes. We develop a Fuzzy-Logic learning algorithm that helps guide users learn how to improve pump parameters for these patients. We recruited and trained 13 nurses on the software and report their improvement in pump administration, basal rates adjustments, and ICR modulation.

## Introduction

The need to increase the capacity and competencies of healthcare workers using innovative and continuous training approaches is of utmost importance in providing necessary care for patients, especially after the ongoing COVID-19 pandemic [1–3]. Diabetes management and insulin pumps have been challenging topics for school nurses, nurses in surgical wards, physicians, and most importantly people with diabetes [4, 5]. The more challenging type-1 diabetes (T1D) demands continuous monitoring, treatment adjustments, meal inspections, and activity patrol. The complexity of the matter increases and becomes even more challenging when dealing with T1D children [6–8]. Nurses must have the proper training and knowledge on utilizing technological advances to keep a tighter control on the more volatile blood sugar levels in children to decrease microvascular complications.

T1D affects an estimated 20 million people worldwide and continues to grow yearly at a 4% rate [9–12]. Several treatments are available for patients ranging from multiple daily injections (MDI) of insulin to continuous subcutaneous insulin infusion (CSII) therapy and sophisticated hybrid and closed-loop systems [13–15]. An estimated 40% of T1D patients, and growing, utilize pumps and sensor technology as an intensive insulin therapy mechanism [16]. Recent advances in the areas of Artificial Pancreas and closed-loop systems are promising to alleviate many of the hurdles and complications associated with managing T1D pumps. Children of all ages with T1D have been switching from MDI to CSII therapies because of improved overall quality of life [5]. CSII pumps provide a greater flexibility in insulin administration and meal planning supplemented by the information retrieved from continuous glucose monitoring devices [2, 6]. The goal of creating fully automated insulin delivery systems will revolutionize glucose management and offer an increased lifestyle flexibility for T1D patients.

People with diabetes are three times more likely to be hospitalized compared to the general population [17–19]. Those with T1D are reported to experience higher rates of complications, longer stays, and increased hospital mortality [20–22]. T1D patients at hospitals often experience high rates of hypoglycemia, several events of hyperglycemia, and ketoacidosis complications [23, 24]. Moreover, they experience difficulties in adjusting insulin doses to accommodate required nutritional support, fasting periods, and modified scheduled insulin therapies [23–25]. The use of T1D pump technologies in the hospital setting has demonstrated the ability to improve patients’ glycemic control, decreasing episodes of hypoglycemia, and provide an improved quality of life [26, 27]. As the popularity of T1D technologies continues to increase, hospital healthcare providers are facing the need to manage the inpatient care of users with pump devices [28].

The demand for specialized diabetes inpatient services is increasing as the global T1D incidence rates continues to grow [11, 23, 29]. However, the lack of awareness and lack of healthcare professionals trained in the use of pump technologies is hindering the required care [16]. Globally, T1D patients find that many hospitals and clinics lack policies and guidelines for managing T1D technologies and lack the expertise and personnel for proper consultation on treatments provided by these technologies. This widespread lack of expertise can lead to medication errors and harmful outcomes for patients [30]. For these reasons, the American Diabetes Association (ADA), the American Association of Clinical Endocrinologists, and the Diabetes Technology Society all advocate allowing the use of insulin pumps at hospitals for patients that are physically and mentally able to operate their devices [31–34]. In addition, they recommend that hospitals put in place policies for the use of these technologies and train their healthcare providers on CSII pump management. They recommend that providers are directly involved in adjusting pump settings and insulin requirements on these devices during patient visits and after discharge [35, 36]. As diabetes educators, trained nurses can assist and support children and families in their transition to pump therapy [37, 38]. Basal rate adjustments, insulin-to-carbohydrate ratio (ICR) regulation, and insulin correction-doses are settings that providers should be able to assist T1D patients with [39].

In this work, we develop a T1D training simulation software for providers to assist with adjusting pump parameters in CSII devices. Existing simulators in the field are designed for developers to test and train algorithms for closed-loop systems [40–42]. Little advance has been made since the release of the basic AIDA simulator for diabetes education in 1996 [43]. The training environment we develop, called Leeno, aims at teaching healthcare professionals how pumps work, how to set pump parameters to improve glycemic control, and how to avoid severe hypoglycemia events in T1D patients. The environment utilizes a set of validated virtual patients that mimic glycemic responses of T1D patients from past clinical trials. To guide training, we develop and validate a Fuzzy-Logic learning algorithm to assist in the adjustment of basal and ICR rates. We recruited 13 nurses from Emergency, In-Patient, and Operation Theatre hospital departments to conduct T1D training sessions on Leeno and report their pre- and post-usage results of the platform. The results indicate an improvement in pump administration, basal rate adjustments, and ICR modulation. Because an increasing number of diabetes patients are using insulin pump technologies, it is of utmost importance that healthcare providers have the knowledge and means to care for these patients.

## Methods

In this section, we outline the work we performed to provide an effective medium to train physicians and nurses on how to operate a CSII T1D pump. We developed an industry-grade software application, called Leeno, to provide users with an electronic dashboard to set pump parameters and simulate their parameter choices on virtual T1D patients. Leeno allows users to run virtual clinical trials to test various pump parameters and patient conditions, effectively providing a training medium for users. The mathematical models governing the virtual patients and their glycemic responses have been verified and clinically validated in one of our previous studies [42]. Leeno was built to accommodate users who have no prior knowledge on how to tweak glycemic parameters to provide better patient outcomes (ex. higher time-in-target values). To accommodate these users, we developed an assisted-learning environment integrated into the Leeno application. The environment utilizes a Fuzzy-Logic algorithm to guide users on pump values they should tweak to improve patient glycemic outcomes in subsequent trials.

### Application framework

We utilized best practices of Object-Oriented design and modular programming in building the Leeno software. The component running the virtual patients was hosted on the cloud, while the code running the pump dashboard interface, software engine to communicate between the virtual patients and simulation view, and the smart algorithm all run on a local personal machine. JavaFX [44] components were used to build a modern, user-friendly interface and support the GUI elements of Leeno. As the software was built using Java, it is available to run on various operating systems including Windows, MacOS, and Linux.

**Fig 1:**
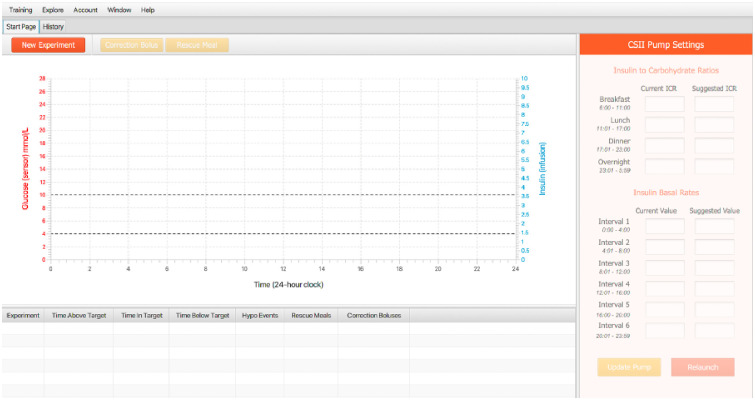
Pump interface view. (Right) Users can specify ICR and insulin basal rate pump settings. (Left) Simulation Results displayed include glucose readings over a 10-minute interval increments and display meal amounts, basal rates, and insulin boluses.

Upon logging into Leeno (see S1 Fig in S1 File), a user is directed to the pump interface page, as shown in Fig 1. The interface is split into two main views: The view on the right of the screen contains pump settings, and the view on the left displays simulation results. The pump settings include fields to input meal-dependent ICR values and six basal rates to be used throughout the day.

Upon login, Leeno pulls virtual patient clinical data from a cloud server and prepares the environment for user simulations. A user can initiate a New Experiment (S2 Fig in S1 File) and supply a meal protocol for a patient (S3 Fig in S1 File). Experiments run for a 48-hour period, plotting the glucose values of the selected virtual patients, along with the insulin dosages administered by the CSII pump calculated from the ICR and basal rates on the pump interface. When the 48-hour simulation is completed, the user is prompted to update the values of the pump. At this stage, the user can analyze the performance of the previous parameters with respect to glucose time-in-target values and hypo- and hyper-glycemia events. The user can then select new parameters and relaunch the experiment for another 48-hour run. Details pertaining to the system design and object-oriented components are outlined in S4-S7 Figs and S1 Text in S1 File for interested readers.

### Smart algorithm

It is generally a difficult process for clinicians and T1D patients to decide what parameters need to change to improve subsequent simulation runs. At the clinic, physicians and nurses observe patients’ previous trends and try to suggest modifications on pump parameters to improve future targets. To assist with this decision-making process, we developed a Fuzzy-Logic learning algorithm, integrated into Leeno, to guide users on potential pump parameters that should be changed. The algorithm acts as an expert system by integrating T1D clinical knowledge into computational rules that can be utilized to better calibrate the pump parameters. Users conducting experiments on the Leeno software can consult this Smart Algorithm after every 48-hour simulation run to guide them on the ICR values and basal rates that can be changed to improve the next 48-hour pump performance.

### Basal rate changes

**Fig 2:**
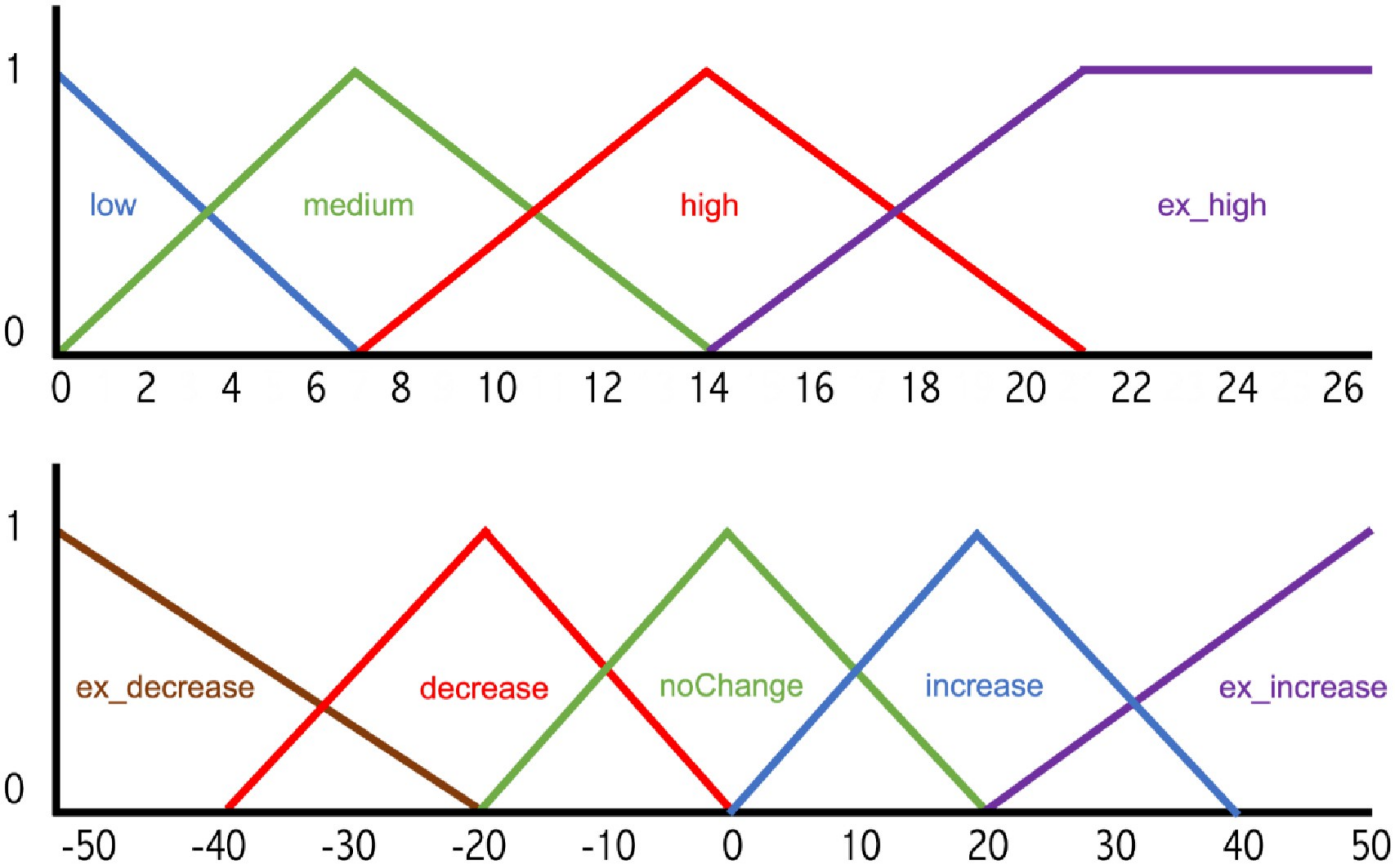
Algorithm membership function. (Top) Input Membership Function. The algorithm classifies glucose input into 4 sets: low, medium, high, and ex_high. The x-axis plots blood glucose values and the y-axis plots the range of truth value from 0 to 1. (Bottom) Output Membership Function involved in the center of gravity defuzzification process to produce basal-rate insulin changes.

As a 48-hour simulation unfolds, the algorithm monitors the patient glucose readings, basal rates administered by the pump, meal amounts, and insulin boluses. The algorithm divides a day into six intervals (4 hours per interval) and compares the glucose trends across intervals in different days. The algorithm’s objective is to find ways to increase glucose time-in-target and minimize hypoglycemia events. Given the outlined input, the algorithm implements a fuzzy-logic method to suggest changes to basal rates in each of the six intervals. The algorithm performs a fuzzification step on the patient glucose readings to classify a given blood glucose numeric value into one or more fuzzy sets and associates a membership score with each set. Fig 2 (Top) displays the membership functions used in the fuzzification process.

During the fuzzification step, the algorithm utilizes a combination of triangular and trapezoidal based input membership functions that transform blood glucose readings of a virtual patient into one (or more) of four defined sets: “low”, “medium”, “high”, and “ex_high” fuzzy sets. The transformation maps a blood glucose reading of 7.0 to the “medium” set with a 100% degree of membership, maps 14.0 to the “high” set with a full membership, and maps a reading of 21 to the “ex_high” set. We defined Values between (0–7.0), (7.0–14.0), and (14.0–21.0) to belong simultaneously to two sets, but with varying degrees of membership as outlined in Fig 2. The membership function computes and decides the most appropriate set(s) a blood glucose reading should belong to. Once this process is complete for the various endpoints of each interval during the 48-hour run, the algorithm proceeds with applying Fuzzy-Logic rules to determine what if any changes in insulin basal rates are necessary.

Our algorithm includes 88 Fuzzy-Logic rules inspired from the clinical knowledge in the literature to determine if the basal rates need to change for any of the six intervals. The 88 rules are assessed for each of the 4-hour intervals and the most appropriate rule is applied using the center of gravity defuzzification method. The following are 5 sample rules used in the fuzzification process. The remaining rules are provided in S1 Table in S1 File:

1. IF (Intv1GlucoseBegin IS low AND Intv1GlucoseEnd IS low AND Intv2GlucoseBegin IS low AND Intv2GlucoseEnd IS low) THEN InsulinRequirement IS noChange.
2. IF (Intv1GlucoseBegin IS low AND Intv1GlucoseEnd IS medium AND Intv2GlucoseBegin IS low AND Intv2GlucoseEnd IS high) THEN InsulinRequirement IS increase.
3. IF (Intv1GlucoseBegin IS low AND Intv1GlucoseEnd IS medium AND Intv2GlucoseBegin IS low AND Intv2GlucoseEnd IS exHigh) THEN InsulinRequirement IS exIncrease.
4. IF (Intv1GlucoseBegin IS exHigh AND Intv1GlucoseEnd IS high AND Intv2GlucoseBegin IS exHigh AND Intv2GlucoseEnd IS high) THEN InsulinRequirement IS decrease.
5. IF (Intv1GlucoseBegin IS exHigh AND Intv1GlucoseEnd IS high AND Intv2GlucoseBegin IS high AND Intv2GlucoseEnd IS low) THEN InsulinRequirement IS exDecrease.

In rule 1, the algorithm compares the glucose fuzzy sets at the beginning and end of a 4-hour interval in day 1 and day 2. If the glucose readings are all low, the algorithm decides not to change the insulin basal rate for that interval. The repeated low glucose readings might be a result of an aggressive bolus prior to entering the interval duration. In rule 2, when the glucose levels increase at the end of the 4-hour interval on both days 1 and 2, the algorithm decides to slightly increase the basal rate associated with this interval to counter the increase of insulin in day 3. In rule 3, the basal rate is increased at a higher rate due to a slightly upward trend in glucose behavior. Rules 4 and 5 exhibit a suggested decrease in basal insulin requirement due to a downward glucose 2-day trend during the same time interval. The bottom part of Fig 2 displays the output membership function used in the defuzzification step to translate the decision regarding whether to increase an insulin basal rate, decrease the rate, or keep it unchanged. Similar to the fuzzification process, the defuzzification step computes and decides the most appropriate output decision. The algorithm will recommend an alteration to the basal rate depending on the selected set and the degree of membership assigned to it. We selected the x-axis bounds for the input and output sets from our observations of past clinical data.

**Fig 3:**
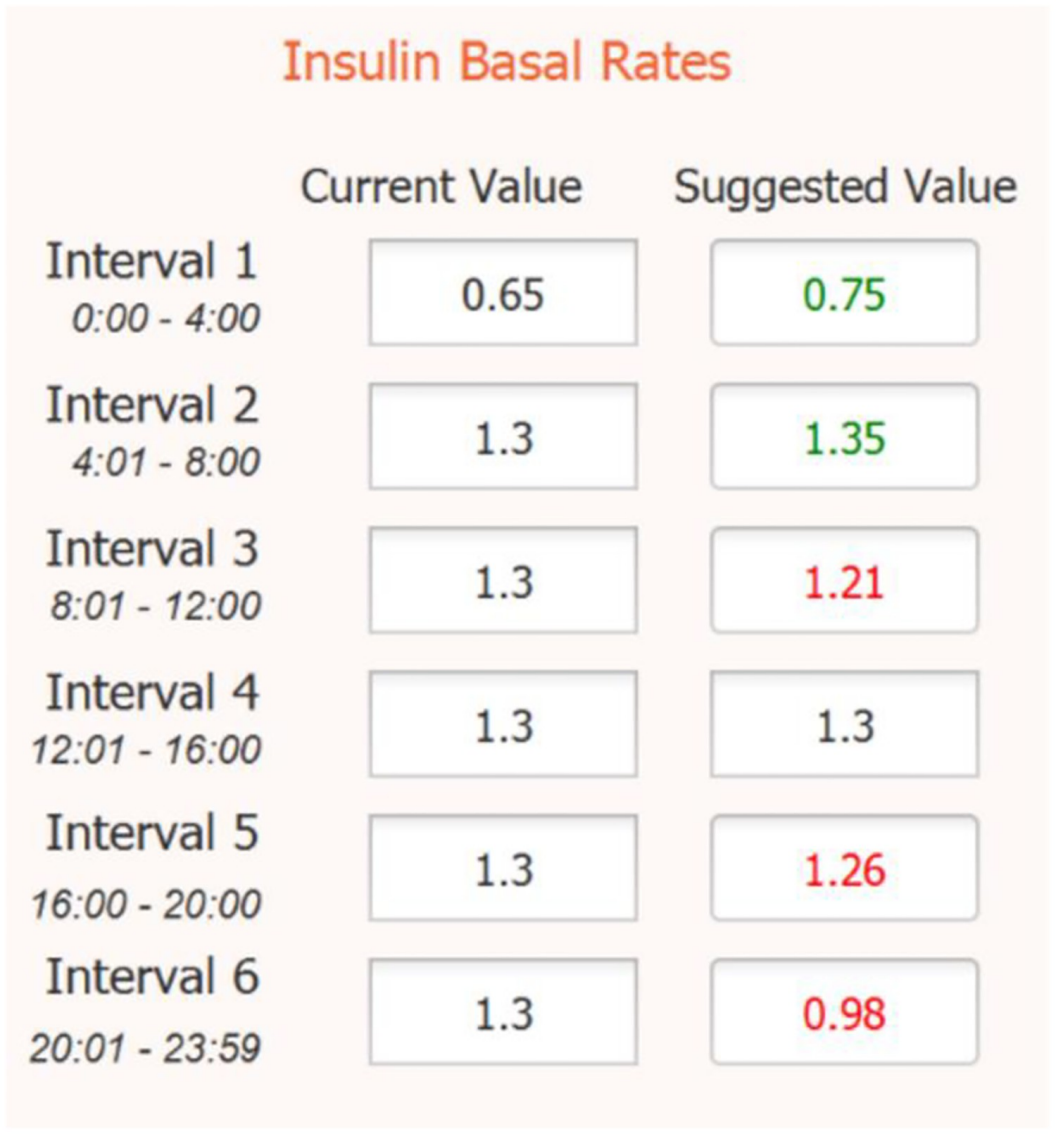
Algorithm insulin basal rate suggestions. Upon analyzing a 48-hour simulation run, the Fuzzy-Logic algorithm returns suggested changes to basal rates. Values displayed in green prompt the user to a necessary increase in the pump parameter, red values suggest decreasing a basal rate, and black values suggest no change.

S1 Algorithm in S1 File outlines the logic used to process the defuzzification results and provide a maximum 20 percent increase or decrease in basal rates, as practiced in various clinical protocols. The new basal rate suggestions are then displayed to the user as shown in Fig 3. To perform the core mathematical fuzzification and defuzzification functions behind the fuzzy logic process, we integrated the jFuzzyLogic [45] control application in our software.

### ICR suggestions

**Fig 4:**
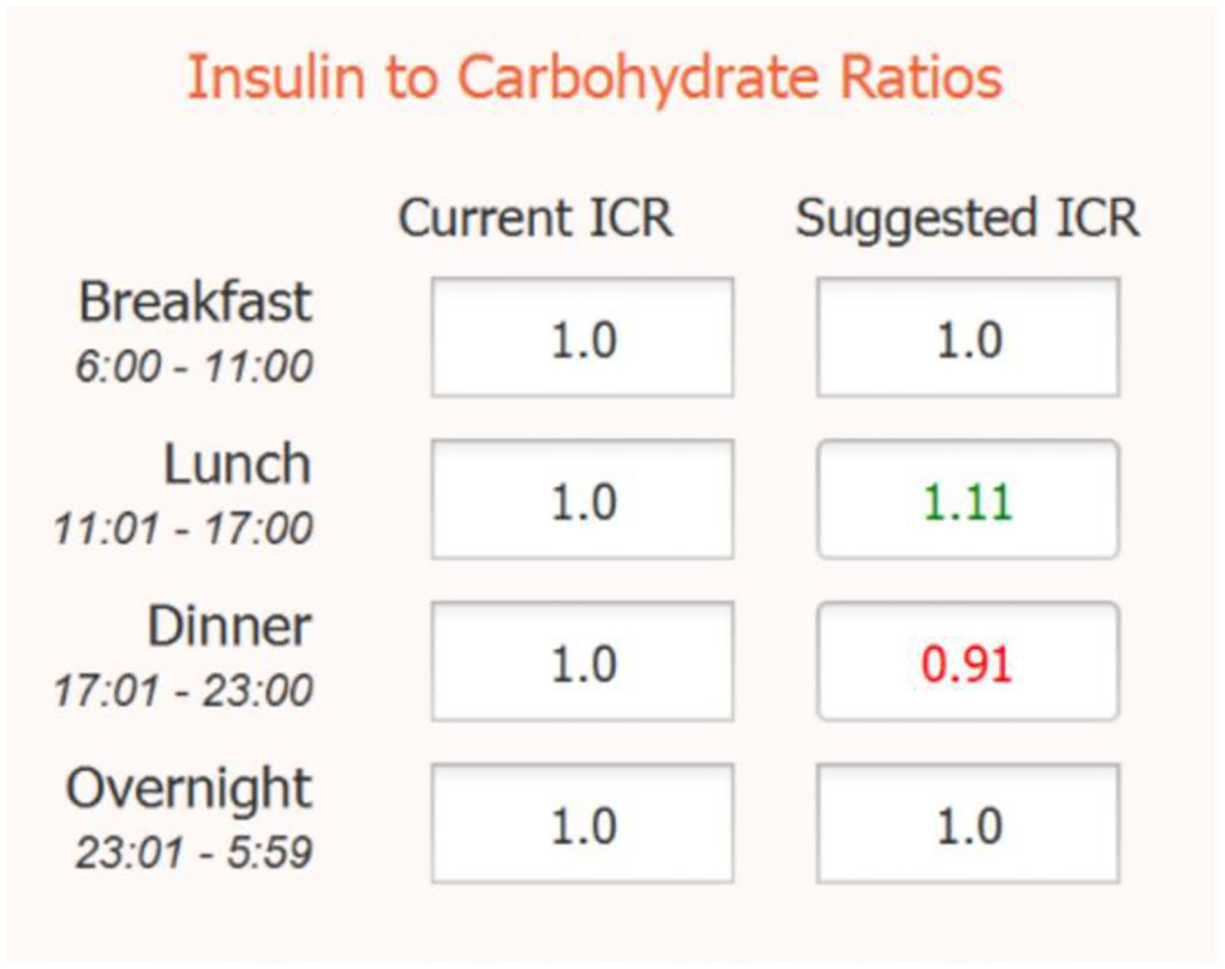
Computed ICR values. The suggested ICR values in green indicate an increase from the current ICR and suggested ICR values in red indicate a decrease from the current ICR.

The algorithm updates meal ICRs separately from basal rates. Meal ICRs are used in calculating insulin boluses that are inherently larger doses of insulin required to counteract glucose changes after meals. The Fuzzy-Logic algorithm monitors glucose values up to 4 hours after each meal. In addition, the algorithm attempts to recompute and recalibrate the different ICR values by comparing the carbohydrate intakes during a meal with the glycemic effect resulting from the given bolus. The calculation is complicated with the existence of multiple meals or snacks during any 4-hour window. To adjust for multiple meals, the algorithm computes time between meals and correlates the first derivative of the glucose trend during and after meals. S2 and S3 Algorithms in S1 File detail the logic used in this process and return the decision of increasing, decreasing, or maintaining the ICR value as is. The values returned are displayed to the user as shown in Fig 4.

We describe in the Results & discussion section some validation results pertaining to our proposed algorithm and its performance on a month-long computational study. In addition, we used the Fuzzy-Logic learning algorithm to perform a study to assess improvements in CSII insulin pump calibration of nurses at a local hospital.

### Ethics statement

The Queen Hospital Ethics Committee approved the computational study (approval no 2021421). All nurse participants gave their written informed consent prior to their inclusion in the study.

## Results & discussion

The aim of developing the Fuzzy-Logic Learning algorithm was to capture as much clinical decision-making rules as possible to provide physicians and users a training environment that helps improve glucose time-in-target for patients and reduce hypoglycemia events. The incremental improvements suggested by the algorithm every 48-hours to basal and ICR values converge to better glycemic control. We present in this section results validating the performance of the algorithm on improving glucose control. In addition, as a translational research effort, we conducted training sessions for thirteen nurses on the Leeno application and outline the improvement they exhibited on glycemic outcomes.

### Smart algorithm performance

We validated the performance of our Fuzzy-Logic algorithm on the T1D simulation development platform for artificial pancreas algorithms framework, Ulna [42]. Ulna provides an in-silico clinical trial environment populated with clinically validated T1D patient models. Our goal using Ulna was to compare our CSII Fuzzy-Logic learning algorithm against a traditional CSII open-loop treatment.

**Fig 5:**
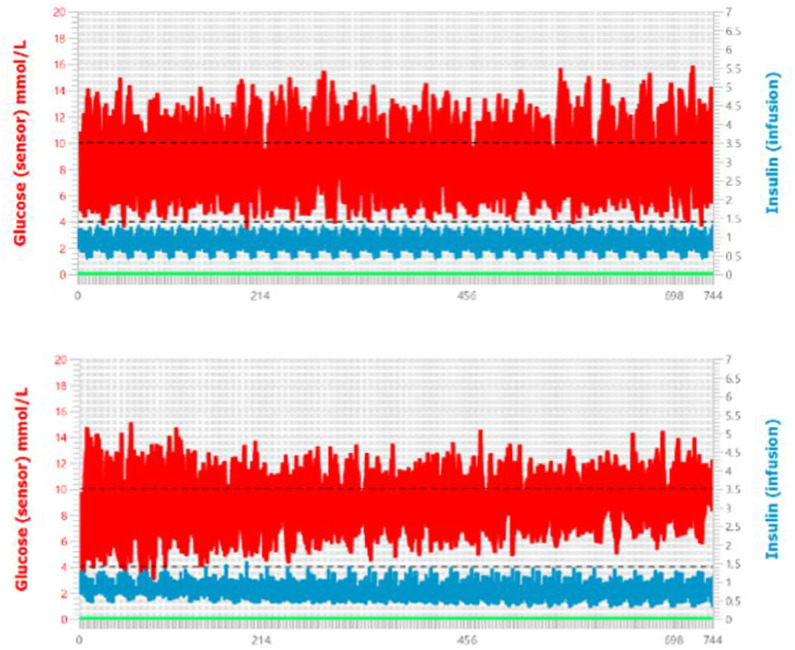
Comparison of Open-Loop vs. Fuzzy-Logic Learning algorithms. (Top) 31-day trial using the Open-Loop Algorithm (bottom) Fuzzy-Logic Learning Algorithm trial results. Red Lines represent the 25% and 75% IQR median range of blood glucose levels across all 15 patients, the blue lines represent insulin basal injections, and the x-axis plots clinical trial simulation time in hours.

We designed our experiments to run for 62 days on 15 adult patients. Using the same meal and variability protocols, we conducted a 31-day trial for each patient using the conventional CSII open loop algorithm implemented in Ulna, and a second 31-day trial using our proposed fuzzy-logic algorithm. The Fuzzy-Logic algorithm was allowed to self-update its basal and ICR values every 48-hours according to its internal S1-S3 Algorithms in S1 File and fuzzy rules in the S1 Table in S1 File. The results of the trials are plotted in Fig 5 and highlighted in Table 1.

**Table 1:** CSII Open-Loop vs CSII Fuzzy-Logic Learning Algorithms comparison for the overall simulation study period of 31 days across the 15 patients.

Outcome	CSII Open-Loop pump therapy (n = 15)	CSII Fuzzy-Logic Learning Algorithm (n = 15)
Time spent at glucose levels (%):		
4.0–10.0 mmol/L*	59% (43–80)	66% (44–79)
< 4.0 mmol/L	2.4% (0.1–16.5)	1.7% (0.1–10.2)
< 3.5 mmol/L	0.7% (0.0–9.5)	1.1% (0.0–6.4)
< 3.1 mmol/L	0.2% (0.0–5.2)	0.5% (0.0–3.7)
> 8.0 mmol/L	53% (22–84)	62% (40–89)
> 10.0 mmol/L	30% (4–56)	29 (14–56)
Hyperglycemia Events, n	76	41
Hypoglycemia Events^$^, n	306	142

Data are median (IQR).

*Primary outcome, defined as 4.0–10.0 mmol/L.

^$^Secondary study outcome.

Our primary outcome was defined to be glucose time-in-target in the range 4.0–10.0 mmol/L. The virtual trial involving the CSII Fuzzy-Logic algorithm resulted in a higher time-in-target outcome compared to the CSII Open-Loop trial (65.9% vs. 58.9%). We defined the secondary outcome of the study to be the number of hypoglycemia and hyperglycemia events. Again, the virtual trial involving the CSII Fuzzy-Logic algorithm outperformed the CSII Open-Loop trial. The open-loop algorithm resulted in 306 hypoglycemia events and 76 hyperglycemia events. The Fuzzy-logic algorithm reduced both events by half. We observed 142 hypoglycemia events across the entire month and 76 hyperglycemia events with our proposed algorithm. The results indicate an overall good incremental improvement with the Fuzzy-Logic learning algorithm. As the algorithm updates its basal and ICR rates every 48-hours by a maximum of 20%, it is expected that the algorithm will not result in drastic volatility. On the contrary, the algorithm is designed to help users tweak existing parameters and safely converge to better results over time. This convergence can be observed in Fig 5 as the interquartile values (IQR) of the glucose readings become tighter and closer to one another over time. In addition to tightening the IQR bounds, the updates that occurred 15 times in the Fuzzy-Logic algorithm throughout the month simultaneously reduced both hypoglycemia and hyperglycemia events. S8 Fig in S1 File expands the plot of the Fuzzy-Logic output in Fig 5 and shows how the divergence from hypoglycemia started to take affect at day 10 onwards. It is important to note that the amount of insulin used at the start and end of the simulation study did not significantly change, rather a better control was achieved by supplying insulin where most necessary. In the first 10 days, 82 hypoglycemia events were observed across the 15 patients, in the next 10 days the number reduced to 38, and in the final 10 days the number of hypoglycemia events was only 22. Although still a CSII algorithm, the Fuzzy-Logic learning component was able to provide a considerable improvement over a conventional CSII treatment. This algorithm can be used as a method to improve the glycemic control of patients on CSII pumps. We believe that the algorithm can be used to train health providers on the pump parameters to update to reduce hypoglycemia events and increase time-in-target values over time.

### Hospital personnel training

The improvements suggested by the algorithm empowered us to conduct training sessions for medical staff at a local hospital. As part of our translational research goals for this work, our training focused on helping medical staff at the hospital use Leeno integrated with the Fuzzy-Logic learning algorithm to calibrate CSII insulin pump parameter values. We recruited thirteen nurses from the In-Patient, Emergency, and Operation Theatre departments for training after obtaining necessary approvals from the Ethics Committee at Queen Hospital. These departments usually host critical patients, some of whom were T1D patients on insulin pumps. All thirteen nurses reported an adequate knowledge of diabetes, but their knowledge of type-1 diabetes pump calibration varied. Three of the nurses reported a moderate knowledge of pump calibration while the remaining ten had basic knowledge.

Training for the nurses was carried out individually and occurred over four stages. In the first stage, nurses were presented with the Leeno application, how virtual simulations worked, what pump parameters were available for a clinical trial, how to launch trials, what data is presented on the interface, and how to read the simulation results. In the second stage, we asked each nurse to run a clinical trial for 1 patient for a duration of 6 days. After each 48-hour mark, the nurse had a chance to update the insulin pump parameters (insulin basal rates and ICRs) to improve time-in-target and reduce hypo and hyperglycemia events. In this stage, nurses did not receive any assistance or suggestions from the learning algorithm. The third stage was the training stage that used the Fuzzy-Logic algorithm on one of the virtual patients with high susceptibility to hypoglycemia. This focused their training on avoiding hypoglycemia events. In this stage, each nurse ran a second clinical trial on a different patient (patient 4) for a duration of 6–24 days with the assistance of our Fuzzy-Logic algorithm. At every 48-hour mark, the algorithm provided suggestions for the nurse on what parameters to change and correlated the suggestions to the rules found in S1 Table and S3 Algorithm in S1 File. We explained the logic behind the algorithm choices at each step to the nurses. The nurses were free to take the suggestion of the algorithm or to override it with their own values. In stage 4, we asked each nurse to re-run the 6-day clinical trial on the same patient they chose in stage 2 and apply the knowledge they gained from the training phase in stage 3. They were not allowed to use the assistance of the Fuzzy-Logic algorithm in this stage. In total, each nurse performed three clinical trials. The outcome of the training sessions is presented in Table 2.

**Table 2:** Training session results for 13 nurses spanning training stages 2–4.

Nurse	Hospital	Pump	Virtual	Training	Outcome (mmol/L)			Hypo	Rescue
No.	Dept	Expert	Patient	Stage	< 4.0	4.0–10.0	> 10.0	Events	Meals
1	In-Patient	Moderate	5	CSII-Prior	23.1	72	4.7	6	200g
			4	Learning AVG	41.8	58.2	0	10	400g
			5	CSII-Post	28.2	71.8	0	9	200g
				ΔCSII	5.1	-0.2	-4.7	3	0g
2	ER	Moderate	5	CSII-Prior	15.7	76.6	7.7	4	125g
			4	Learning AVG	39.7	60.3	0	9	350g
			5	CSII-Post	13.9	86.1	0	3	100g
				ΔCSII	-1.8	9.5	-7.7	-1	-25g
3	ER	Low	5	CSII-Prior	35.5	64.5	0	8	300g
			4	Learning AVG	40.5	59.5	0	9	325g
			5	CSII-Post	5.2	69.9	24.8	2	25g
				ΔCSII	-30.3	5.4	24.8	-6	-275g
4	OT	Low	5	CSII-Prior	8.4	87.8	3.8	3	75g
			4	Learning AVG	43.6	56.4	0	11	375g
			5	CSII-Post	6.6	82.5	10.8	2	25g
				ΔCSII	-1.8	-5.3	7	-1	-50g
5	ER	Moderate	5	CSII-Prior	12.2	62.9	24.8	3	75g
			4	Learning AVG	40.8	59.2	0	9	400g
			5	CSII-Post	9.1	65.7	25.2	2	50g
				ΔCSII	-3.1	2.8	0.4	-1	-25g
6	OT	Low	5	CSII-Prior	14.7	76.6	8.7	5	75g
			4	Learning AVG	41.8	58.2	0	10	375g
			5	CSII-Post	12.6	77.3	10.1	3	75g
				ΔCSII	-2.1	0.7	1.4	-2	0g
7	In-Patient	Low	5	CSII-Prior	12.6	78.7	8.7	5	75g
			4	Learning AVG	42.5	57.5	0	12	350g
			5	CSII-Post	15	51.7	33.2	5	50g
				ΔCSII	2.4	-27	24.5	0	-25g
8	OT	Low	5	CSII-Prior	15	78	7	4	125g
			4	Learning AVG	46.3	53.7	0	11	350g
			5	CSII-Post	9.8	83.2	7	3	75g
				ΔCSII	-5.2	5.3	0	-1	-50g
9	In-Patient	Low	5	CSII-Prior	17.8	73.4	8.7	4	100g
			4	Learning AVG	42.9	57.1	0	9	400g
			5	CSII-Post	8	78.7	13.3	3	50g
				ΔCSII	-9.8	5.3	4.6	-1	-50g
10	ER	Low	5	CSII-Prior	12.9	82.9	4.2	4	100g
			4	Learning AVG	43.3	56.6	0	10	350g
			5	CSII-Post	8	87.8	4.2	3	50g
				ΔCSII	-4.9	4.9	0	-1	-50g
11	OT	Low	5	CSII-Prior	14	78	8	5	100g
			4	Learning AVG	44.9	55.1	0	10	375g
			5	CSII-Post	7.3	66.8	25.9	2	50g
				ΔCSII	-6.7	-11.2	17.9	-3	-50g
12	OT	Low	5	CSII-Prior	15.7	78	6.3	5	100g
			4	Learning AVG	41.8	58.2	0	9	400g
			5	CSII-Post	16.1	55.6	28.3	6	100g
				ΔCSII	0.4	-22.4	22	1	0g
13	ER	Low	5	CSII-Prior	13.6	79.4	7	5	100g
			4	Learning AVG	38.7	61.3	0	12	350g
			5	CSII-Post	14.7	78.3	7	4	100g
				ΔCSII	1.1	1.1	0	-1	0g

Stage 2 results are reported under the CSII-Prior Training Stage, Stage 3 results are under Learning AVG, and CSII-Post report results of Stage 4. ΔCSII reports the difference in performance between Stages 2 and 4. Negative results in Hypo events define improvement performance.

To keep the clinical setting variability as low as possible, each nurse performed their Stage 3 training on Virtual Patient 4, and their Stage 2 and Stage 4 trial on Virtual Patient 5. During the study, when the patient’s glucose level fell below 3, an automatic 25g rescue meal was administered to bring the patient out of hypoglycemia. We define ΔCSII in Table 2 to be the difference in numerical results between outcomes in Stage 2 (CSII-Prior) and Stage 4 (CSII-Post). The results show that 10 out of the 13 nurses were able to reduce hypoglycemia events at Stage 4, post training, by an average of 23%. The time spent < 4.0 mmol/l was reduced from 16% to 11%, a 27% reduction across the study. This reduction in hypoglycemia reduced the number and quantity of rescue meals that the patients needed by 38%. The time-in-target 4.0–10.0 mmol/l outcome was reduced by 3% in the study overall from 76% to 74%. The time > 10.0 mmol/l almost doubled and increased by 91% across the study from 8% to 15%.

The focused training on avoiding hypoglycemia resulted in a respectable decrease of hypo events and times below 4.0 mmol/l. Although this resulted in an increase in time spent in hyperglycemia, it is an understandable consequence of T1D CSII pump management. We focused our study on training the nurses to prioritize avoiding hypoglycemia in their hospital setting. All 13 nurses reported benefiting from training with the learning algorithm and reported a stronger confidence in calibrating pump parameters to improve care for type 1 diabetes patients.

## Conclusion and future work

Managing T1D pumps is an ordeal for most patients. The number of patients on CSII pumps is significant globally and will remain so for years to come until further technology advances are made with the fully automated Artificial Pancreas. Many hospitals, schools, and clinics host patients with CSII devices who require medical attention and continuous adjustment of pump parameters. In this work, we developed a training simulation software, called Leeno, to assist nurses, physicians, and licensed caretakers understand how to best tweak pump parameters to avoid severe hypoglycemia events and keep glucose within an acceptable range for as long as possible. The 88 rules we coded into the Fuzzy-Logic learning algorithm used by some clinicians in adjusting basal and ICR rates for T1D patients was able to considerably reduce hypoglycemia and improve time-in-target values when applied on a set of 15 virtual patients. In the hospital setting, the algorithm and the Leeno package were able to help the nurses improve their T1D glucose management skills. With further training and more time spent on exploring and studying the choices of the Fuzzy-Logic algorithm, we believe that medical staff at hospitals, schools, and community centers can significantly get better at calibrating insulin pump parameters, hence improving care for T1D patients and assisting in times of critical need. The modular design of the application allows for different algorithms to be plugged into the system and made available for training.

## Supporting information

S1 File This file containing S1-S8 Figs, S1 Text, S1-S3 Algorithms, and S1 Table. (PDF)

## References

[1] World Health Organization, *Transforming and scaling up health professionals’ education and training*, in *World Health Organization Guidelines* 2013. 2013, WHO. 26042324

[2] Yeh T., Yeung M., and Mendelsohn Curanaj F.A., Managing Patients with Insulin Pumps and Continuous Glucose Monitors in the Hospital: to Wear or Not to Wear. Current diabetes reports, 2021. 21(2): p. 7–7. doi: 10.1007/s11892-021-01375-7 33449214 PMC7810103

[3] Zhou W., et al., Long-term training in diabetes-related knowledge, attitudes, and self-reported practice among diabetes liaison nurses. The Journal of international medical research, 2020. 48(2): p. 300060519882838–300060519882838. 10.1177/0300060519882838 PMC7873921 31662018

[4] Bierschbach J.L., Cooper L., and Liedl J.A., Insulin pumps: what every school nurse needs to know. J Sch Nurs, 2004. 20(2): p. 117–23. doi: 10.1177/10598405040200021201 15040760

[5] Sweeney T.J., Kenny D.J., and Schubert C.C., Inpatient Insulin Pump Therapy: Assessing the Effectiveness of an Educational Program. Journal for Nurses in Professional Development, 2013. 29(2). 10.1097/NND.0b013e318286c5da 23657039

[6] Abdullah N., et al., Management of insulin pump therapy in children with type 1 diabetes. Arch Dis Child Educ Pract Ed, 2014. 99(6): p. 214–20. doi: 10.1136/archdischild-2013-304501 25125555

[7] Wheeler B.J., et al., Insulin pump-associated adverse events in children and adolescents—a prospective study. Diabetes Technol Ther, 2014. 16(9): p. 558–62. 24796368 10.1089/dia.2013.0388

[8] Cooper M.N., et al., A population-based study of risk factors for severe hypoglycaemia in a contemporary cohort of childhood-onset type 1 diabetes. Diabetologia, 2013. 56(10): p. 2164–70. 23832082 10.1007/s00125-013-2982-1

[9] Imperatore G., et al., Projections of type 1 and type 2 diabetes burden in the U.S. population aged <20 years through 2050: dynamic modeling of incidence, mortality, and population growth. Diabetes Care, 2012. 35(12): p. 2515–20. 23173134 10.2337/dc12-0669 PMC3507562

[10] Tuomilehto J., The emerging global epidemic of type 1 diabetes. Curr Diab Rep, 2013. 13(6): p. 795–804. 24072479 10.1007/s11892-013-0433-5

[11] Sayers A., et al., Evidence for a persistent, major excess in all cause admissions to hospital in children with type-1 diabetes: results from a large Welsh national matched community cohort study. BMJ Open, 2015. 5(4): p. e005644. 10.1136/bmjopen-2014-005644 PMC4420955 25869680

[12] Maahs D.M., et al., Epidemiology of type 1 diabetes. Endocrinol Metab Clin North Am, 2010. 39(3): p. 481–97. 20723815 10.1016/j.ecl.2010.05.011 PMC2925303

[13] Peyser T., et al., The artificial pancreas: current status and future prospects in the management of diabetes. Ann N Y Acad Sci, 2014. 1311: p. 102–23. doi: 10.1111/nyas.12431 24725149

[14] Hanazaki K., et al., Current topics in glycemic control by wearable artificial pancreas or bedside artificial pancreas with closed-loop system. J Artif Organs, 2016. 19(3): p. 209–18. doi: 10.1007/s10047-016-0904-y 27142278

[15] Lind M., et al., Sustained Intensive Treatment and Long-term Effects on HbA1c Reduction (SILVER Study) by CGM in People With Type 1 Diabetes Treated With MDI. Diabetes Care, 2021. 44(1): p. 141–149. doi: 10.2337/dc20-1468 33199470

[16] Umpierrez G.E. and Klonoff D.C., Diabetes Technology Update: Use of Insulin Pumps and Continuous Glucose Monitoring in the Hospital. Diabetes Care, 2018. 41(8): p. 1579–1589. doi: 10.2337/dci18-0002 29936424 PMC6054505

[17] Donnan P.T., et al., Hospitalizations for people with type 1 and type 2 diabetes compared with the nondiabetic population of Tayside, Scotland: a retrospective cohort study of resource use. Diabetes Care, 2000. 23(12): p. 1774–9. doi: 10.2337/diacare.23.12.1774 11128351

[18] Icks A., et al., Hospitalization among diabetic children and adolescents and the general population in Germany. German Working Group for Pediatric Diabetology. Diabetes Care, 2001. 24(3): p. 435–40. doi: 10.2337/diacare.24.3.435 11289464

[19] Tomlin A.M., et al., Hospital admissions in diabetic and non-diabetic patients: a case-control study. Diabetes Res Clin Pract, 2006. 73(3): p. 260–7. doi: 10.1016/j.diabres.2006.01.008 16504336

[20] Gamble J.M., et al., Admission hypoglycemia and increased mortality in patients hospitalized with pneumonia. Am J Med, 2010. 123(6): p. 556 e11–6. doi: 10.1016/j.amjmed.2009.11.021 20569764

[21] Umpierrez G.E., et al., Hyperglycemia: an independent marker of in-hospital mortality in patients with undiagnosed diabetes. J Clin Endocrinol Metab, 2002. 87(3): p. 978–82. doi: 10.1210/jcem.87.3.8341 11889147

[22] Viens N.A., et al., Role of diabetes type in perioperative outcomes after hip and knee arthroplasty in the United States. J Surg Orthop Adv, 2012. 21(4): p. 253–60. 23327852 10.3113/jsoa.2012.0253

[23] Mendez C.E. and Umpierrez G.E., Management of Type 1 Diabetes in the Hospital Setting. Curr Diab Rep, 2017. 17(10): p. 98. doi: 10.1007/s11892-017-0919-7 28913745

[24] Cryer P.E., Hypoglycemia in type 1 diabetes mellitus. Endocrinol Metab Clin North Am, 2010. 39(3): p. 641–54. doi: 10.1016/j.ecl.2010.05.003 20723825 PMC2923455

[25] Gingras V., et al., Impact of erroneous meal insulin bolus with dual-hormone artificial pancreas using a simplified bolus strategy—A randomized controlled trial. Sci Rep, 2018. 8(1): p. 2621. doi: 10.1038/s41598-018-20785-4 29422651 PMC5805693

[26] Writing Group for the, D.E.R.G., et al., Association between 7 years of intensive treatment of type 1 diabetes and long-term mortality. JAMA, 2015. 313(1): p. 45–53. doi: 10.1001/jama.2014.16107 25562265 PMC4306335

[27] Gingras V., et al., Treatment of mild-to-moderate hypoglycemia in patients with type 1 diabetes treated with insulin pump therapy: are current recommendations effective? Acta Diabetol, 2018. 55(3): p. 227–231. doi: 10.1007/s00592-017-1085-8 29224132

[28] Cook C.B., et al., Management of inpatient hyperglycemia: assessing perceptions and barriers to care among resident physicians. Endocr Pract, 2007. 13(2): p. 117–24. doi: 10.4158/EP.13.2.117 17490924

[29] Umpierrez G.E., et al., Management of hyperglycemia in hospitalized patients in non-critical care setting: an endocrine society clinical practice guideline. J Clin Endocrinol Metab, 2012. 97(1): p. 16–38. doi: 10.1210/jc.2011-2098 22223765

[30] Investigators N.-S.S., et al., Hypoglycemia and risk of death in critically ill patients. N Engl J Med, 2012. 367(12): p. 1108–18. doi: 10.1056/NEJMoa1204942 22992074

[31] American Diabetes Association Professional Practice, C., et al., 16. Diabetes Care in the Hospital: Standards of Medical Care in Diabetes-2022. Diabetes Care, 2022. 45(Suppl 1): p. S244–S253. doi: 10.2337/dc22-S016 34964884

[32] Funnell M.M., et al., National standards for diabetes self-management education. Diabetes Care, 2012. 35 Suppl 1: p. S101–8. doi: 10.2337/dc12-s101 22187467 PMC3632167

[33] Grunberger G., et al., American Association of Clinical Endocrinologists and American College of Endocrinology 2018 Position Statement on Integration of Insulin Pumps and Continuous Glucose Monitoring in Patients with Diabetes Mellitus. Endocr Pract, 2018. 24(3): p. 302–308. doi: 10.4158/PS-2017-0155 29547046

[34] Wallia A., et al., Consensus Statement on Inpatient Use of Continuous Glucose Monitoring. J Diabetes Sci Technol, 2017. 11(5): p. 1036–1044. doi: 10.1177/1932296817706151 28429611 PMC5950996

[35] Houlden R.L. and Moore S., In-hospital management of adults using insulin pump therapy. Can J Diabetes, 2014. 38(2): p. 126–33. doi: 10.1016/j.jcjd.2014.01.011 24690507

[36] Noschese M.L., et al., Patient outcomes after implementation of a protocol for inpatient insulin pump therapy. Endocr Pract, 2009. 15(5): p. 415–24. doi: 10.4158/EP09063.ORR 19491071

[37] Swift P.G., Diabetes education in children and adolescents. Pediatr Diabetes, 2009. 10 Suppl 12: p. 51–7. 19754618 10.1111/j.1399-5448.2009.00570.x

[38] Lange K., et al., ISPAD Clinical Practice Consensus Guidelines 2014. Diabetes education in children and adolescents. Pediatr Diabetes, 2014. 15 Suppl 20: p. 77–85. doi: 10.1111/pedi.12187 25182309

[39] AbdulAziz Y.H., et al., Insulin pump initiation and education for children and adolescents—a qualitative study of current practice in New Zealand. Journal of diabetes and metabolic disorders, 2019. 18(1): p. 59–64. doi: 10.1007/s40200-019-00390-6 31275875 PMC6582118

[40] Wilinska M.E., et al., Simulation environment to evaluate closed-loop insulin delivery systems in type 1 diabetes. J Diabetes Sci Technol, 2010. 4(1): p. 132–44. doi: 10.1177/193229681000400117 20167177 PMC2825634

[41] Man C.D., et al., The UVA/PADOVA Type 1 Diabetes Simulator: New Features. J Diabetes Sci Technol, 2014. 8(1): p. 26–34. doi: 10.1177/1932296813514502 24876534 PMC4454102

[42] Smaoui M.R., Rabasa-Lhoret R., and Haidar A., Development platform for artificial pancreas algorithms. PLoS One, 2020. 15(12): p. e0243139. doi: 10.1371/journal.pone.0243139 33332411 PMC7746189

[43] Lehmann E.D., Experience with the Internet release of AIDA v4.0—http://www.diabetic.org.uk.aida.htm—an interactive educational diabetes simulator. Diabetes Technol Ther, 1999. 1(1): p. 41–54. doi: 10.1089/152091599317567 11475304

[44] Weaver J.L., *JavaFX Script*: *Dynamic Java Scripting for Rich Internet/Client-side Applications*. 1st edition ed. FirstPress. 2007, Place of publication not identified: Apress L P.

[45] Cingolani P. and Alcala-Fdez J., jFuzzyLogic: a Java Library to Design Fuzzy Logic Controllers According to the Standard for Fuzzy Control Programming. International Journal of Computational Intelligence Systems, 2013. 6: p. 61–74.

