# Supplementary material for: Leeno: Type 1 diabetes management training environment using smart algorithms
Source: PLoS One. 2022 Sep 15;17(9):e0274534. doi: 10.1371/journal.pone.0274534 (PMC9477299; doi:10.1371/journal.pone.0274534)
Supplement: S1 File — (PDF) [file pone.0274534.s001.pdf]

SUPPLEMENTARY MATERIAL

Figures

- **S1 Figure: Login Interface to Leeno**. Users with an account can login on their personal machines. (Page 2)
- **S2 Figure: Launching a New Experiment**. A user can select from a range of virtual patients. (Page 2)
- **S3 Figure: Meal Protocol selection**. A user can select a CSII T1D experiment protocol including 3 meals and snacks. (Page 3)
- **S4 Figure: Leeno's class diagram**. (Page 4)
- **S5 Figure: BodyController diagram**. (Page 5)
- **S6 Figure: Engine class diagram**. (Page 6)
- **S7 Figure: Simulation tab displaying a two days simulation graph with the amount of carbohydrates for each meal**. (Page 7)
- **S8 Figure: Fuzzy-Logic Learning Algorithm Simulation Study**. (Page 8)

Methods:

- **S1 Text: System Design Overview** (Page 9)

Algorithms

- **S1 Algorithm: basalVerdict()**. This algorithm determines if a basal rate should increase, decrease, or not change at a subsequent 48-hour run. (Page 11)
- **S2 Algorithm: ICRVerdict()**. This algorithm inspects the current ICR value and follows a set of rules to determine if changing the ICR will produce a more stable glucose trend. (Page 13)
- **S3 Algorithm: analyzeICRPerformance()**. This algorithm analyzes the effect of an insulin bolus on the glucose trend within a 4-hour window from having a meal. (Page 16)

Tables

- **S1 Table: Fuzzy-Logic Rules Used to determine changes in insulin basal rates of CSII Pumps**. (Page 18)

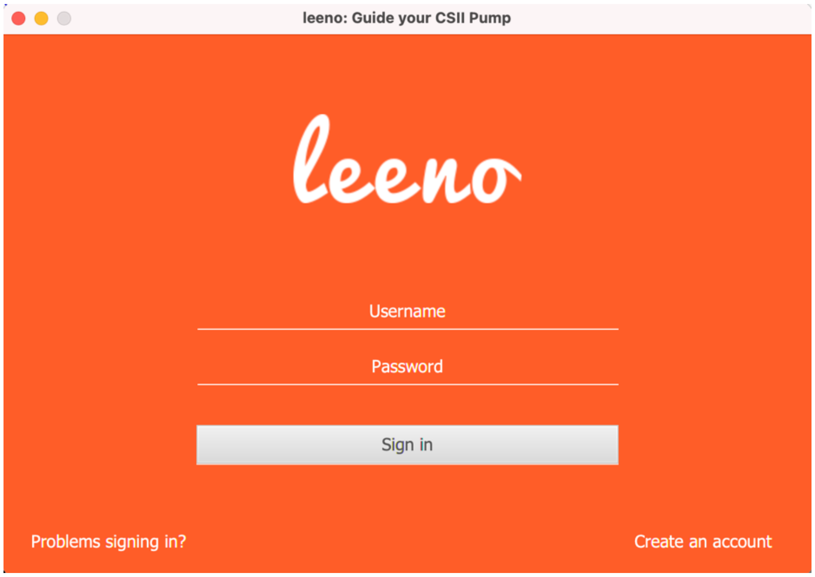

S1 Figure: Login Interface to Leeno. Users with an account can login on their personal machines.

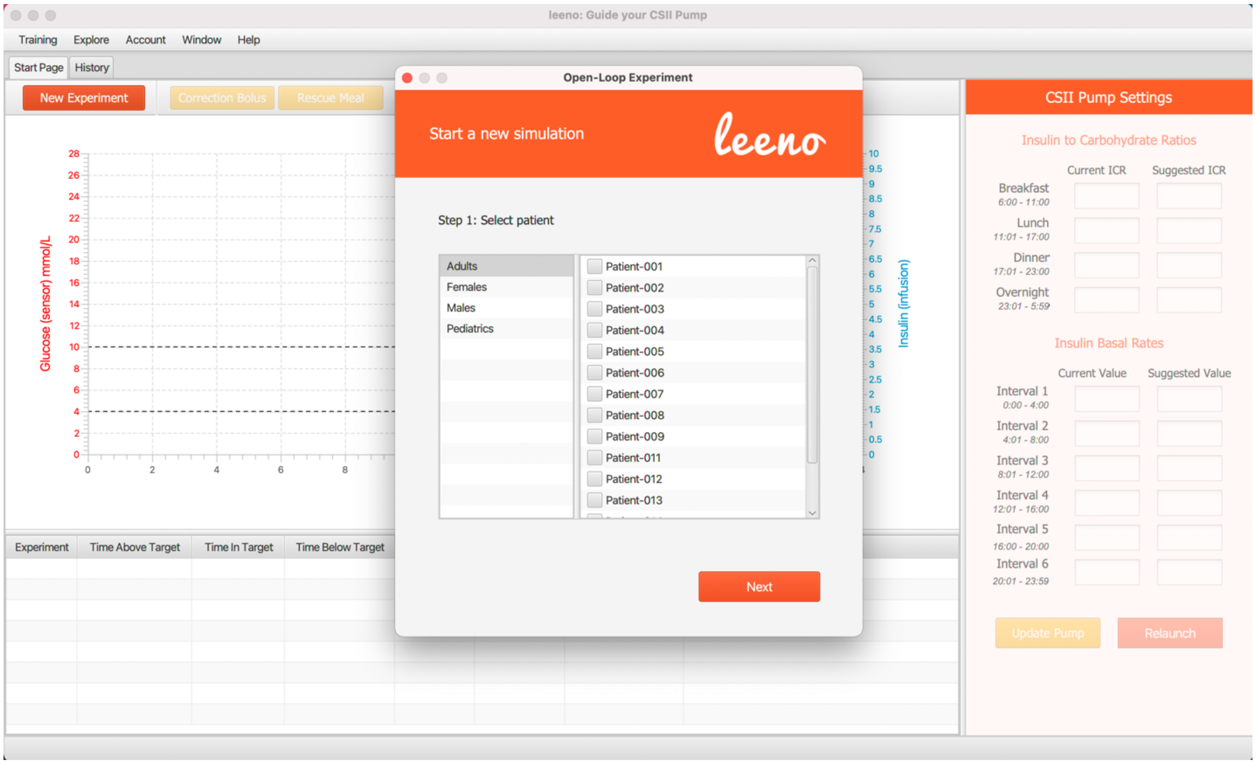

S2 Figure: Launching a New Experiment. A user can select from a range of virtual patients.

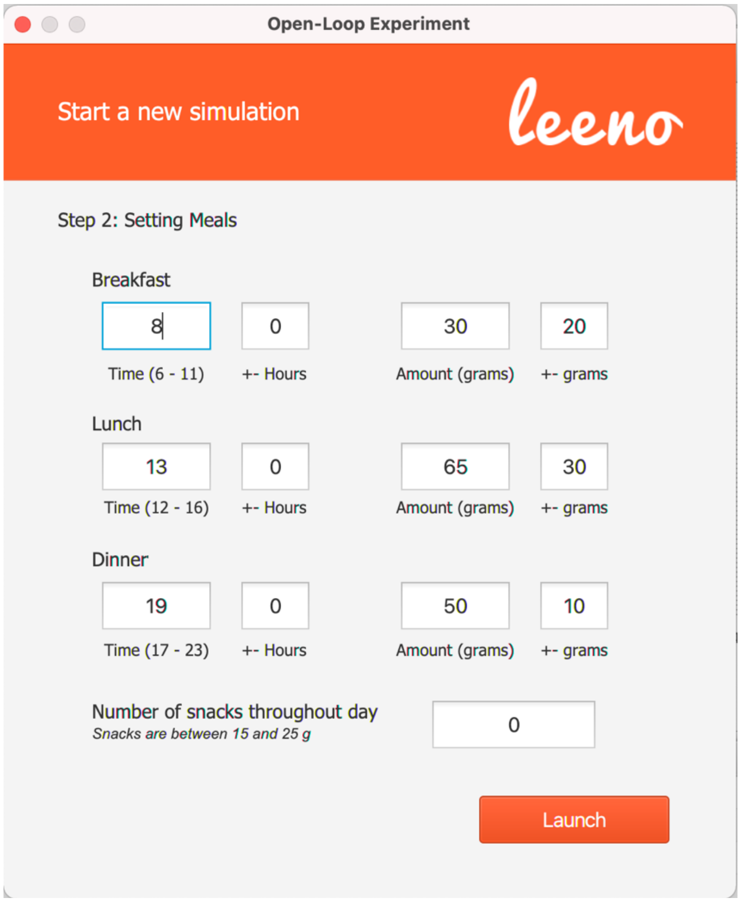

S3 Figure: Meal Protocol selection. A user can select a CSII T1D experiment protocol including 3 meals and snacks. Each meal can be scheduled within hours specified by the user and include a varying carbohydrate amount.

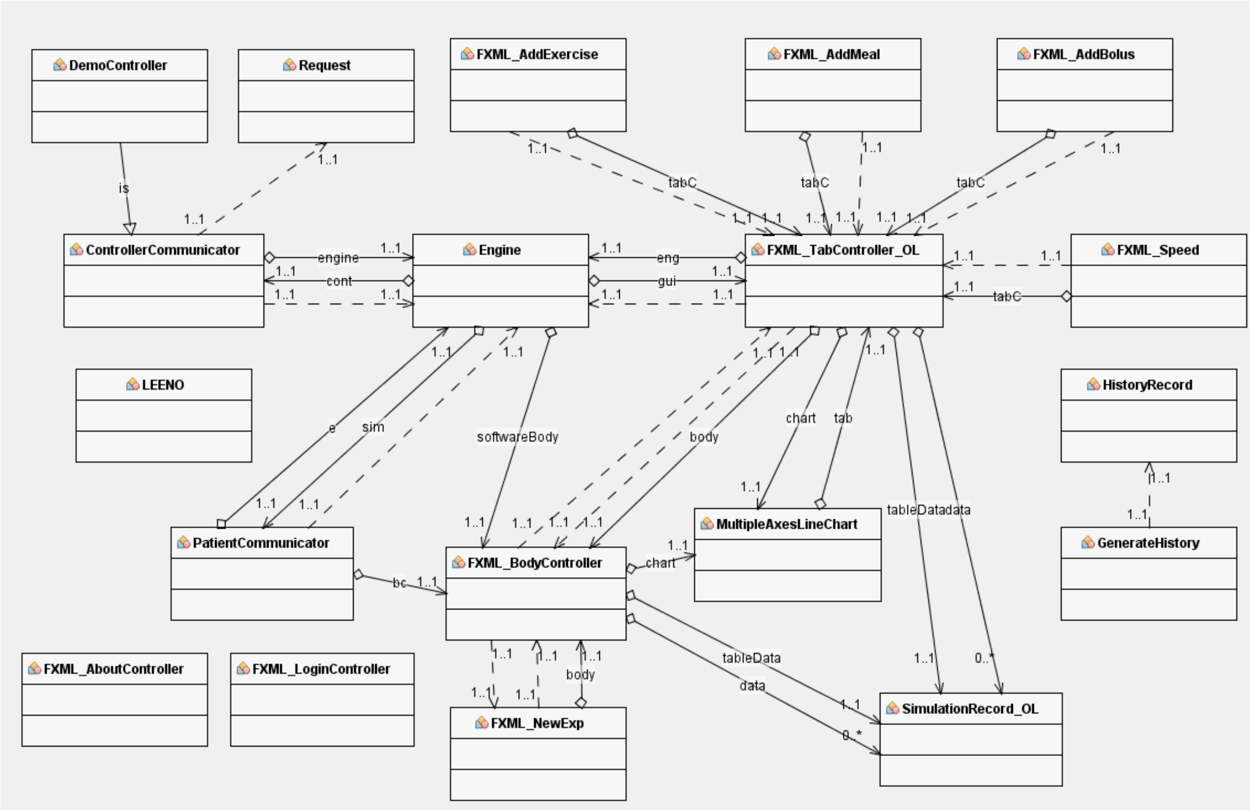

S4 Figure: Leeno's class diagram. The central classes are the `FXML_BodyController` which sets up the application for a new simulation, the `Engine` class that is connected with the `ControlCommunicator` and `PatientCommunicator` classes to run the simulation on a selected patient, and `FXML_TabController_OL` class which creates a new tab for every new simulation.

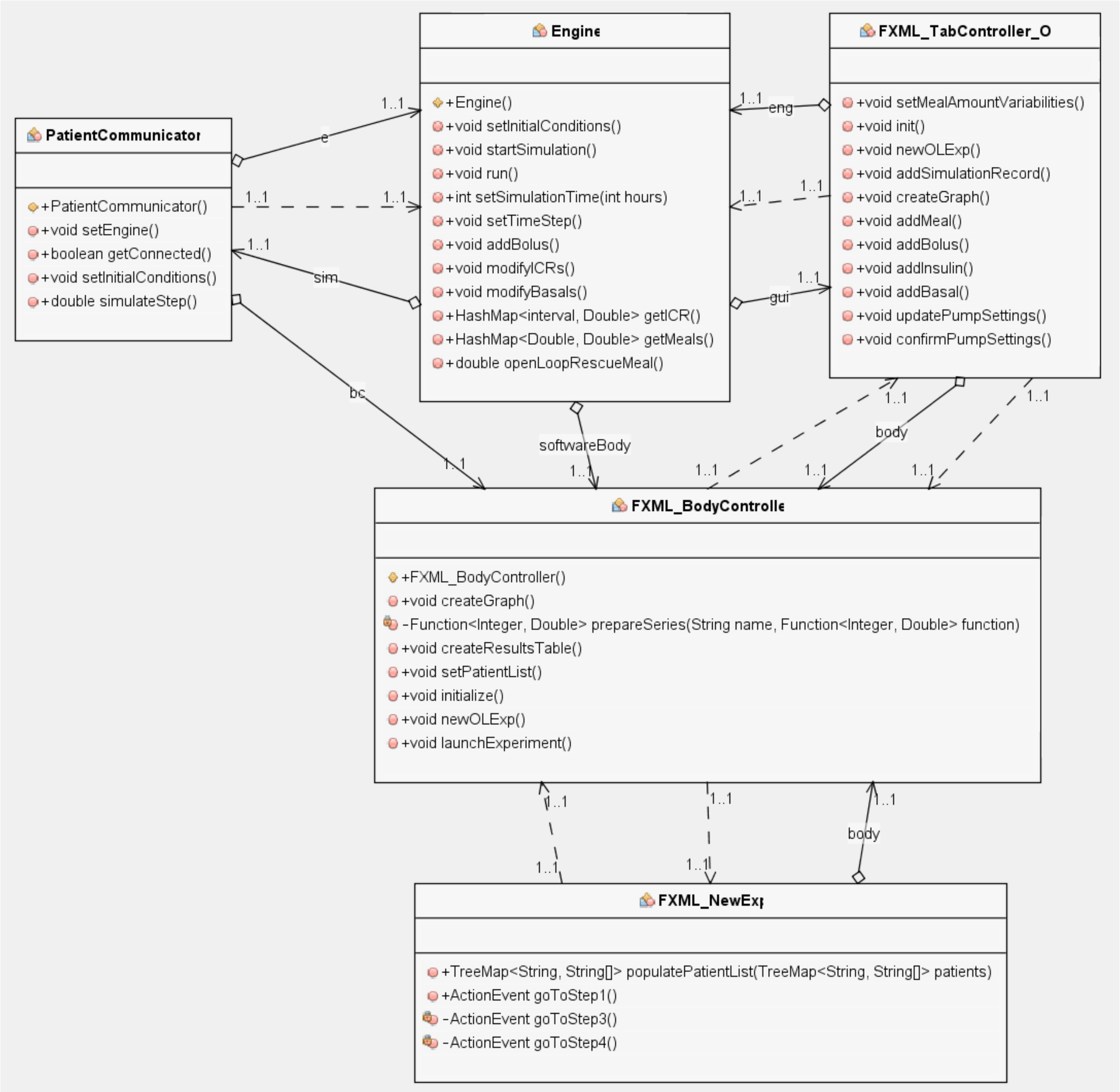

S5 Figure: BodyController diagram. `FXML_BodyController` is responsible for setting up the application for a new simulation, initializing the virtual patient by creating an object of `PatientCommunicator` class to connect with patient server, and initializes the simulation `Engine`.

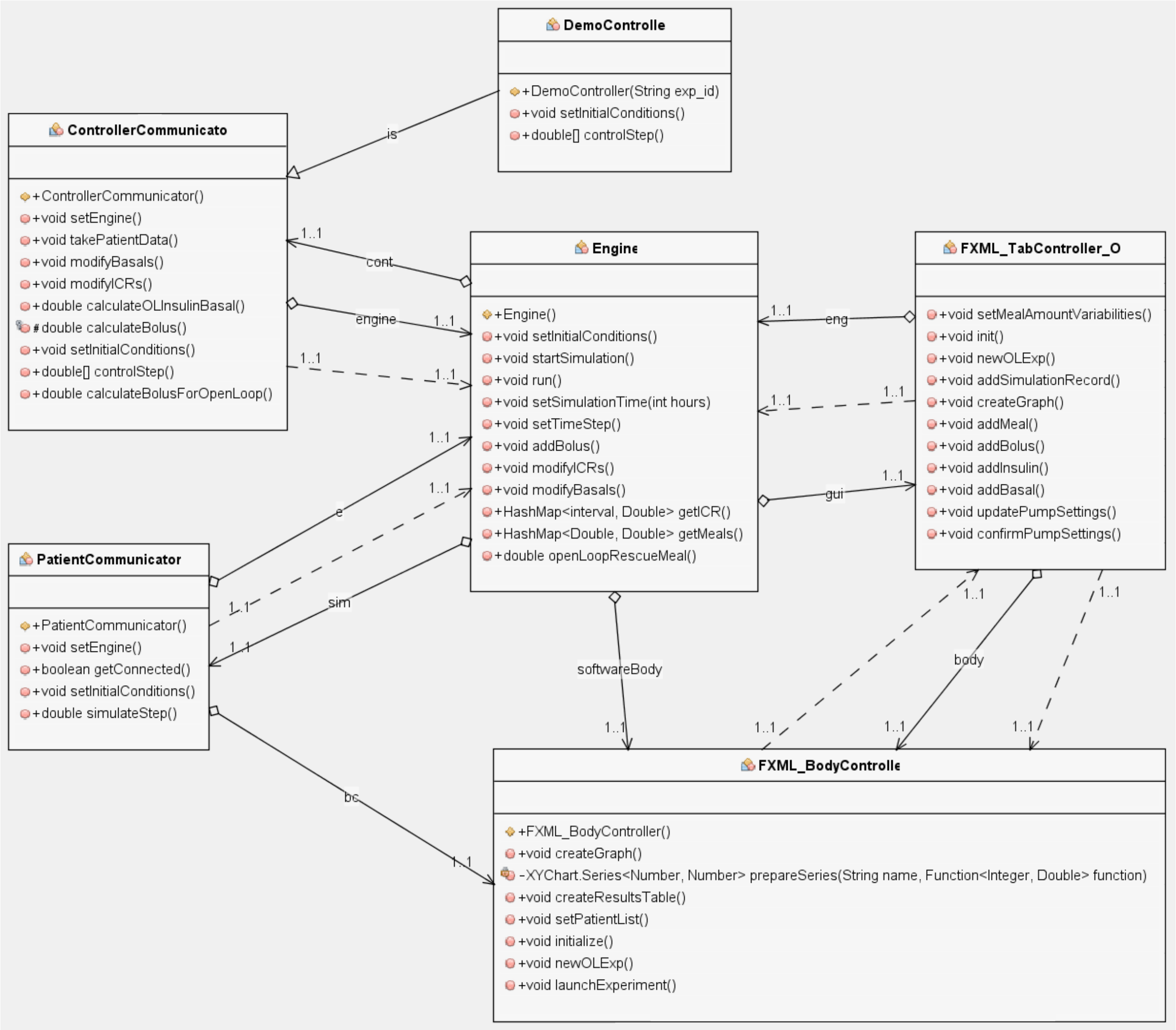

S6 Figure: Engine class diagram. The `Engine` class is responsible for running the simulation, retrieving the patient's glucose level from `PatientCommunicator`, retrieving insulin calculated doses from `ControllerCommunicator`, and sending the results to `FXML_TabController_OL` to be plotted on the user's view.

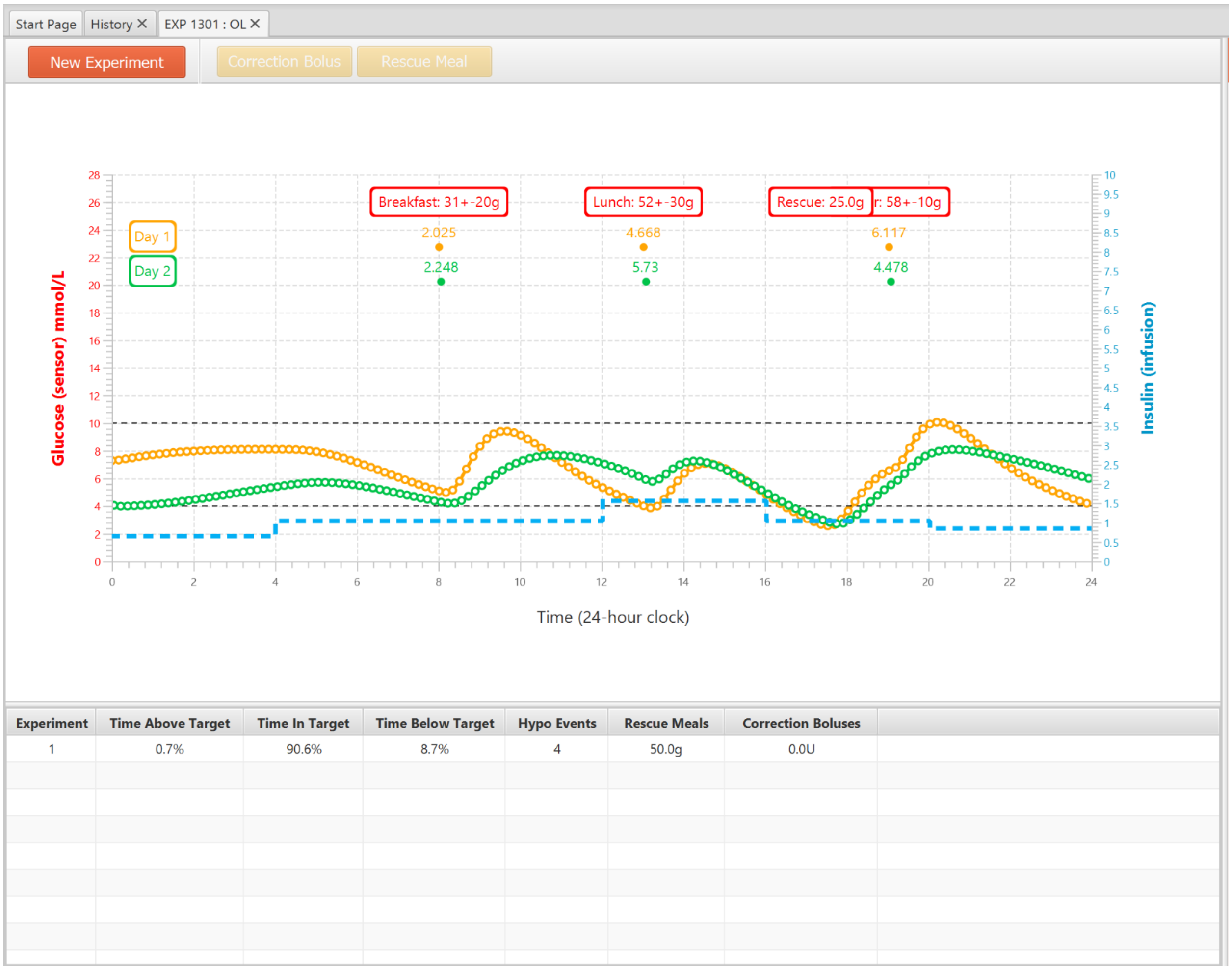

Experiment	Time Above Target	Time In Target	Time Below Target	Hypo Events	Rescue Meals	Correction Boluses
1	0.7%	90.6%	8.7%	4	50.0g	0.0U

S7 Figure: Simulation tab displaying two days consisting of meals, insulin bolus doses (bullet points under each meal), 25g carbohydrates rescue snacks, basal rates (blue dashed line), and the final simulation result at the bottom panel.

(a)

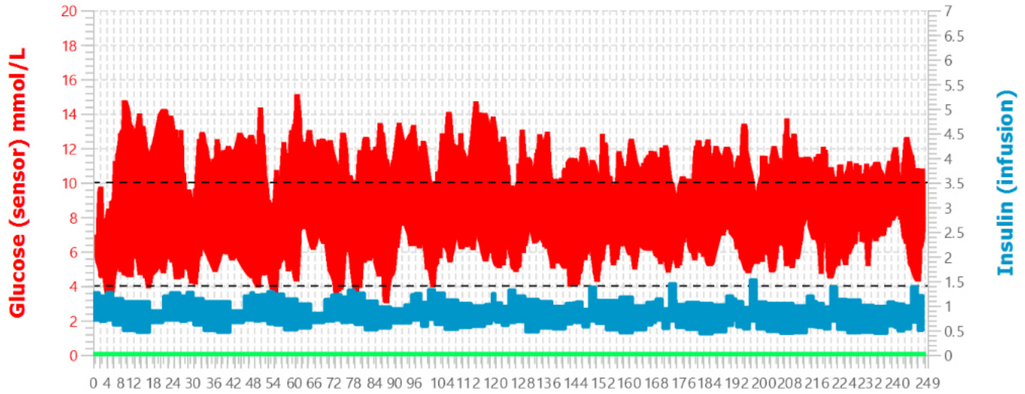

(b)

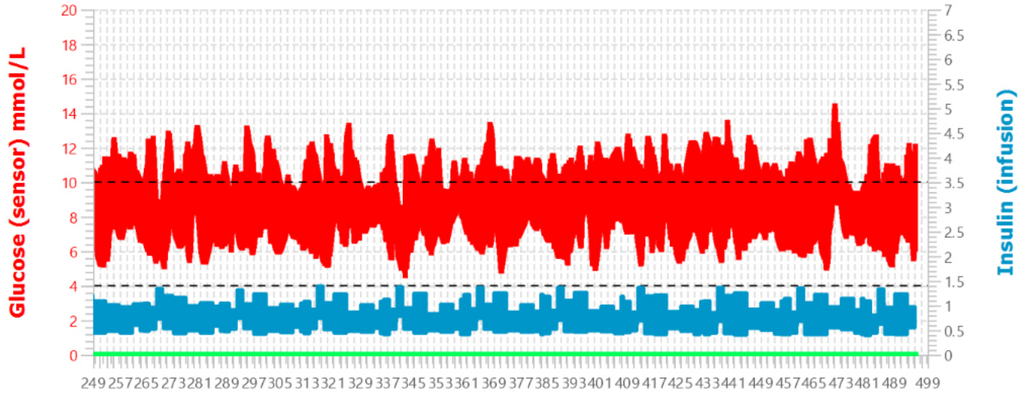

(c)

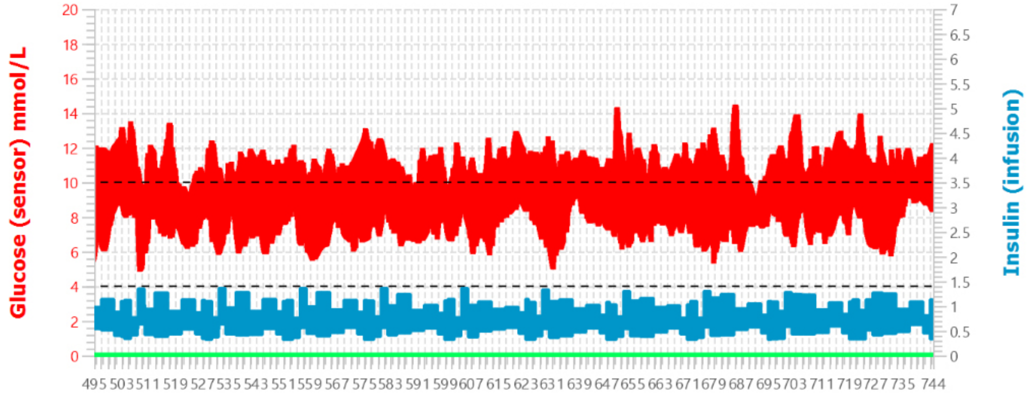

S8 Figure: Fuzzy-Logic Learning Algorithm Simulation Study. (a) Plots the first 248 hours (~10 days) and results in 82 hypoglycemia events and a 61.4% time-in-target. Figure (b) plots the next 10 days and results in 38 hypoglycemia events and an accumulative 62.7% time-in-target. Figure (c) displays the last 10 days of the study which resulted in 22 hypoglycemia events and a an accumulative 65.9% time in target.

S1 Text: System Design Overview

Upon the application's launch, Leeno connects to the cloud and retrieves virtual patient clinical data from a cloud server. This data will be used to run the training simulations of virtual diabetes patients. The data has been collected from real T1D patients from one of our past studies and consists of the following parameters:

1. Age, BMI, HBA1c %, Duration of diabetes
2. Total insulin daily dose
3. Breakfast ICR
4. Lunch ICR
5. Dinner ICR
6. Overnight ICR
7. Open loop pump parameters containing a list of 48 basal rates for every half an hour during the day
8. Ordinary Differential Equation (ODE) parameters that dictate insulin and glucose absorption, sensitivities, and responses.

Leeno is built using a Model-View-Controller (MVC) design pattern. The main view of Leeno has a controller class named `FXML_BodyController` which instantiates objects of the `FXML_TabController_OL`, `MultipleAxesLineChart`, `SimulationRecord_OL`, `FXML_NewExp`, `PatientCommunicator`, and `Engine` classes. The `FXML_BodyController` contains methods to launch a new experiment (Figure S5), Some of those methods are `createGraph()` and `prepareSeries()` which create the graph and the glucose points to be plotted on the graph during the simulation. The method named `newOLExp()` connects this class with `FXML_NewExp` class to populate the patients for the user. Method `goToStep1()` in class `FXML_NewExp` calls the method `populatePatientList()` that will list all virtual patients in a chickListView to be displayed in a window for the user to choose a single patient from the list and run the simulation on.

When the user clicks on launch, the method `goToStep4()` is called. This method will make sure the user entered all the proper data for the simulation to be launched, then calls the method `launchExperiment()` in the `FXML_BodyController` class, which initializes the algorithm controller and send all the selected patient data to the controller. The algorithm controller is an object of class `DemoController` that runs an open loop algorithm to set basal rates and insulin boluses to the patient during the simulation. `launchExperiment()` also creates and initializes a new simulation tab in the GUI with the simulation duration, start time, time step, and patient glucose level.

Then `launchExperiment()` will initialize the virtual patient by creating an object of `PatientCommunicator` class to connect with patient server, and initializes the simulation `Engine`.

The application engine class (Figure S6) then runs the open loop simulation using the method `run()` on the selected patient in the new tab that has been created with a simulation ID. This method has a loop that allow the simulation to run for 2 days (48 hours). In each iteration, the `run()` method will get the insulin basal of the current time step by calling `calculateOLInsulinBasal()` method in `ControlCommunicator` class, checking if the patient is having a meal or not and how much carbohydrate in that meal, then calls the `controlStep()` method in the `DemoController` class passing to it the current time, current glucose level, and amount of carbohydrates if any. The `controlStep()` method calls `calculateBolusForOpenLoop()` to calculate the dosage of insulin bolus based on the current time value and corresponding meal ICR and return it to to the caller. This open loop algorithm is simple and does not take into consideration the blood glucose response of the T1D patients after the meal, it just calculates insulin bolus doses based on patients' ICR values. The algorithm that we develop in this paper uses a smart fuzzy-logic learning algorithm that takes into consideration T1D patients' glucose responses and suggests new basal rates and ICR values which helps reducing hypoglycemia events and increasing time in target.

Method `run()` will resume by calling `openLoopRescueMeal()` to give the patient a 25g carbohydrate snack in case of severe hypoglycemia. After that, `addSimulationRecord()` in class `FXML_TabController_OL` is invoked to plot on the simulation graph the patient's glucose level, the insulin basal rate, the amount of carbohydrate intake in case of a meal, the corresponding insulin bolus of that meal, rescue meals in case of severe hypoglycemia, and correction insulin bolus in case of severe hyperglycemia. Also in each time step, a table below the simulation graph is being updated with the percentage of the time above targeted glucose level (10 mmol/L), the percentage of the time in targeted glucose level between 4 mmol/L and 10 mmol/L, the percentage of the time below targeted glucose level (4 mmol/L), number of hypoglycemia events, total grams of rescue meals, total amount of correction bolus. Finally, the `run()` method will call the `simulateStep()` method in class `PatientCommunicator` passing to it insulin basal, insulin bolus, and the carbohydrate amount if any to get the new patient glucose level for the next iteration.

After a simulation of 48-hours, a user can call our Fuzzy-Logic algorithm implemented in the class `SmartController` that inherits from the `ControllerCommunicator` to suggest new basal rates and new ICR values. These new suggested values after each simulation run can assist users in understanding how tweak the pump parameters to improve a patients' glucose response. Running the simulation with our learning algorithm iteratively for several times has shown to help physicians and nurses train themselves on effectively changing pumps' parameters. Hence, Leeno supplied with our learning algorithm can provide training for users to enhance their decision making process and help them get better at calibrating pump parameters, and in turn, improve care for T1D patients.

S1 Algorithm:
basalVerdict (*fuzzyInsulinRequirement, previousBasal, CHO, currentAvgBasal, maxBasal, minBasal*)

```
   // CASE 1: No trend was found in data for time period
   if fuzzyInsulinRequirement == −1 then
      return previousBasal
   end if

   // CASE 2: Insulin Basal Modification Required ++
   if fuzzyInsulinRequirement > 20 then
      meal1 ← false
      meal2 ← false
      for each amount in CHO do
         if amount > 0 in this interval in Day 1 then
            meal1 ← true
         end if
         if amount > 0 in this interval in Day 2 then
            meal2 ← true
         end if
      end for

      if meal1 and meal2 then return previousBasal     // Will decide on insulin amount with ICR
                                                       // calculation
      else
         // Update basal rate by max rate: 20%
         // basal rate cannot be 1.5 times the average basal rate
         // new basal cannot be 20% higher than the max basal rate during day
         newBasal ← previousBasal * 1.2
         newBasal ← min(1.5 * currentAvgBasal, newBasal)
         newBasal ← min(1.2 * maxBasal, newBasal)
         return newBasal
      end if

   end if

   // CASE 3: Insulin Bolus Modification Required --
   if fuzzyInsulinRequirement < −20 then
      meal1 ← false
      meal2 ← false
      for each amount in CHO do
         if amount > 0 in this interval in Day 1 then
            meal1 ← true
         end if
```

```
        if amount > 0 in this interval in Day 2 then
            meal2 ← true
        end if
    end for
    if meal1 and meal2 then return previousBasal    // Will decide on insulin amount with ICR
                                                    // calculation
    else
        //The new basal rate cannot also be less than 20% of lowest basal rate during day
        newBasal ← (previousBasal − (previousBasal * 0.2) )
        newBasal ← max(newBasal, currentAvgBasal / 1.5)
        newBasal ← max(minBasal − (minBasal * 0.2), newBasal)
        return newBasal
    end if
end if

// CASE 4: Update Basal Rate between 0% and 20%
newBasal ← (previousBasal + previousBasal * fuzzyInsulinRequirement /100)

// any basal rate cannot be 1.5 times the average basal rate
newBasal ← min(1.5 * currentAvgBasal, newBasal)

// any basal rate cannot be less 1/1.5 the average basal rate
newBasal ← max(newBasal, currentAvgBasal / 1.5)

// The new basal rate cannot also be less than 20% of lowest basal rate during day
newBasal ← max(minBasal − (minBasal * 0.2), newBasal)

// The new basal rate cannot be more than 20% higher than the max basal rate during day
newBasal ← min(1.2 * maxBasal, newBasal)

return newBasal
```

S2 Algorithm

ICRVerdict(*currentICR*):

```
change ← 1
lessICR ← currentICR
lessICR ← (1.0 / (lessICR / 10)) + change
lessICR ← (1.0 / lessICR) * 10

moreICR ← currentICR
moreICR ← (1.0 / (moreICR / 10)) − change
moreICR ← (1.0 / moreICR) * 10

lessICRMeals ← getMealsGivenWith(lessICR)
moreICRMeals ← getMealsGivenWith(moreICR)
currentICRMeals ← getMealsGivenWith(currentICR)

for each meal in currentICRMeals do
   decision ← analyzeICRPerformance(meal)
   if decision == -2 then          // no decision
      currentICRMeals.remove(meal)
end for

for each meal in lessICRMeals do
   decision ← analyzeICRPerformance(meal)
   if decision == -2 then          // no decision
      lessICRMeals.remove(meal)
end for

for each meal in moreICRMeals do
   decision ← analyzeICRPerformance(meal)
   if decision == -2 then          // no decision
      moreICRMeals.remove(meal)
end for

currentICRMealChange ← median(currentICRMeals)
lessICRMealChange ← median(lessICRMeals)
moreICRMealChange ← median(moreICRMeals)

// Decide whether to select lessICR, currentICR, or moreICR

if lessICRMealChange == 0 and currentICRMealChange == 0 and moreICRMealChange == 0 then
   return currentICR
if lessICRMealChange == 0 and currentICRMealChange == 0 and moreICRMealChange > 0 then
   return currentICR
```

```
if lessICRMealChange == 0 and currentICRMealChange == 0 and moreICRMealChange < 0 then
    return currentICR
if lessICRMealChange == 0 and currentICRMealChange > 0 and moreICRMealChange == 0 then
    return moreICR
if lessICRMealChange == 0 and currentICRMealChange > 0 and moreICRMealChange > 0 then
    return moreICR
if lessICRMealChange == 0 and currentICRMealChange > 0 and moreICRMealChange < 0 then
    return currentICR
if lessICRMealChange == 0 and currentICRMealChange < 0 and moreICRMealChange == 0 then
    return lessICR
if lessICRMealChange == 0 and currentICRMealChange < 0 and moreICRMealChange > 0 then
    return lessICR
if lessICRMealChange == 0 and currentICRMealChange < 0 and moreICRMealChange < 0 then
    return lessICR
if lessICRMealChange > 0 and currentICRMealChange == 0 and moreICRMealChange == 0 then
    return currentICR
if lessICRMealChange > 0 and currentICRMealChange == 0 and moreICRMealChange > 0 then
    return moreICR
if lessICRMealChange > 0 and currentICRMealChange == 0 and moreICRMealChange < 0 then
    return currentICR
if lessICRMealChange > 0 and currentICRMealChange > 0 and moreICRMealChange == 0 then
    return moreICR
if lessICRMealChange > 0 and currentICRMealChange > 0 and moreICRMealChange > 0 then
    return moreICR
if lessICRMealChange > 0 and currentICRMealChange > 0 and moreICRMealChange < 0 then
    return currentICR
if lessICRMealChange > 0 and currentICRMealChange < 0 and moreICRMealChange == 0 then
    return lessICR
if lessICRMealChange > 0 and currentICRMealChange < 0 and moreICRMealChange > 0 then
    return currentICR
if lessICRMealChange > 0 and currentICRMealChange < 0 and moreICRMealChange < 0 then
    return lessICR
if lessICRMealChange < 0 and currentICRMealChange == 0 and moreICRMealChange == 0 then
    return currentICR
if lessICRMealChange < 0 and currentICRMealChange == 0 and moreICRMealChange > 0 then
    return currentICR
if lessICRMealChange < 0 and currentICRMealChange == 0 and moreICRMealChange < 0 then
    return lessICR
if lessICRMealChange < 0 and currentICRMealChange > 0 and moreICRMealChange == 0 then
    return currentICR
if lessICRMealChange < 0 and currentICRMealChange > 0 and moreICRMealChange > 0 then
    return currentICR
if lessICRMealChange < 0 and currentICRMealChange > 0 and moreICRMealChange < 0 then
    return currentICR
```

```
if lessICRMealChange < 0 and currentICRMealChange < 0 and moreICRMealChange == 0 then
    return lessICR
if lessICRMealChange < 0 and currentICRMealChange < 0 and moreICRMealChange > 0 then
    return currentICR
if lessICRMealChange < 0 and currentICRMealChange < 0 and moreICRMealChange < 0 then
    return lessICR

return currentICR
```

S3 Algorithm:
analyzeICRPerformance(*meal*):

```
    lastGlucoseValue = meal.glucoseValueAt4Hours
    // if the tmax is low, we'll use the 3 hour cut, else we keep it at 4 hour cut
    if 4 – 2 * meal.maxGlucoseValueTime > 1 then
            lastGlucoseValue = meal.glucoseValueAt3Hours

    // Check to see if ICR needs to be increased
    if lastGlucoseValue – meal.startGlucoseValue > 1 then

      // Case 1: Glucose is high because there was subsequent meal(s). Do nothing:
      if (meal.hasMealAfter) then
        if meal.mealAfterTime < 180 then
          // a second meal is within 3 hours of current meal, can't really decide, do nothing
           return −2             // no decision
        else
           if meal.mealAfterTime > 180 then
             if meal.glucoseValueAt3Hours − meal.startGlucoseValue > 1.5 then
               return 1          // increase
             else
               return 0          // Keep same

      else     // No meal after this one in 4 hour span
        if meal.startGlucoseValue ≤ 5 then
          // if the meal response is an increase yet the starting glucose value is in hypo, we are
          // not sure what the change should be
          return −2
        if meal.maxGlucoseValue - meal.startGlucoseValue ≤ 2 then
           // if the amplitude is low, we might not need to increase ICR.
           return −2

        return 1

    else
        if meal.lastGlucoseValue − meal.startGlucoseValue < 1 then
           // This could occur because of correction bolus
           if meal.startGlucoseValue > 10 then
             return 0

           return −1     // decrease

   return 0
```

Algorithm 3 returns the decision to increase, decrease or maintain an ICR value after analyzing its meal response. A return value of -1 signals a requirement to decrease ICR, 0 to maintain ICR, 1 to increase ICR and -2 no decision. After the decision is made for each of the three meal responses list, the decisions are returned then stored in *lessICRMealChange*, *moreICRMealChange*, and *moreICRMealChange* lists. The median value is calculated from each of lists to be used in the 27 cases in **Algorithm 2** to decide whether to increase, decrease, or keep the current ICR.

S1 Table: Fuzzy-Logic Rules Used to determine changes in insulin basal rates of CSII Pumps.

1. IF (Intv1GlucoseBegin IS low AND Intv1GlucoseEnd IS low AND Intv2GlucoseBegin IS low AND Intv2GlucoseEnd IS low) THEN InsulinRequirement IS noChange.
2. IF (Intv1GlucoseBegin IS low AND Intv1GlucoseEnd IS low AND Intv2GlucoseBegin IS medium AND Intv2GlucoseEnd IS medium) THEN InsulinRequirement IS noChange.
3. IF (Intv1GlucoseBegin IS low AND Intv1GlucoseEnd IS low AND Intv2GlucoseBegin IS high AND Intv2GlucoseEnd IS high) THEN InsulinRequirement IS noChange.
4. IF (Intv1GlucoseBegin IS low AND Intv1GlucoseEnd IS low AND Intv2GlucoseBegin IS ex_high AND Intv2GlucoseEnd IS ex_high) THEN InsulinRequirement IS noChange.
5. IF (Intv1GlucoseBegin IS medium AND Intv1GlucoseEnd IS medium AND Intv2GlucoseBegin IS low AND Intv2GlucoseEnd IS low) THEN InsulinRequirement IS noChange.
6. IF (Intv1GlucoseBegin IS medium AND Intv1GlucoseEnd IS medium AND Intv2GlucoseBegin IS medium AND Intv2GlucoseEnd IS medium) THEN InsulinRequirement IS noChange.
7. IF (Intv1GlucoseBegin IS medium AND Intv1GlucoseEnd IS medium AND Intv2GlucoseBegin IS high AND Intv2GlucoseEnd IS high) THEN InsulinRequirement IS noChange.
8. IF (Intv1GlucoseBegin IS medium AND Intv1GlucoseEnd IS medium AND Intv2GlucoseBegin IS ex_high AND Intv2GlucoseEnd IS ex_high) THEN InsulinRequirement IS noChange.
9. IF (Intv1GlucoseBegin IS high AND Intv1GlucoseEnd IS high AND Intv2GlucoseBegin IS low AND Intv2GlucoseEnd IS low) THEN InsulinRequirement IS noChange.
10. IF (Intv1GlucoseBegin IS high AND Intv1GlucoseEnd IS high AND Intv2GlucoseBegin IS medium AND Intv2GlucoseEnd IS medium) THEN InsulinRequirement IS noChange.
11. IF (Intv1GlucoseBegin IS high AND Intv1GlucoseEnd IS high AND Intv2GlucoseBegin IS high AND Intv2GlucoseEnd IS high) THEN InsulinRequirement IS noChange.
12. IF (Intv1GlucoseBegin IS high AND Intv1GlucoseEnd IS high AND Intv2GlucoseBegin IS ex_high AND Intv2GlucoseEnd IS ex_high) THEN InsulinRequirement IS noChange.
13. IF (Intv1GlucoseBegin IS ex_high AND Intv1GlucoseEnd IS ex_high AND Intv2GlucoseBegin IS low AND Intv2GlucoseEnd IS low) THEN InsulinRequirement IS noChange.
14. IF (Intv1GlucoseBegin IS ex_high AND Intv1GlucoseEnd IS ex_high AND Intv2GlucoseBegin IS medium AND Intv2GlucoseEnd IS medium) THEN InsulinRequirement IS noChange.

15. IF (Intv1GlucoseBegin IS ex_high AND Intv1GlucoseEnd IS ex_high AND Intv2GlucoseBegin IS high AND Intv2GlucoseEnd IS high) THEN InsulinRequirement IS noChange.
16. IF (Intv1GlucoseBegin IS ex_high AND Intv1GlucoseEnd IS ex_high AND Intv2GlucoseBegin IS ex_high AND Intv2GlucoseEnd IS ex_high) THEN InsulinRequirement IS noChange.
17. IF (Intv1GlucoseBegin IS low AND Intv1GlucoseEnd IS medium AND Intv2GlucoseBegin IS low AND Intv2GlucoseEnd IS medium) THEN InsulinRequirement IS increase.
18. IF (Intv1GlucoseBegin IS low AND Intv1GlucoseEnd IS medium AND Intv2GlucoseBegin IS low AND Intv2GlucoseEnd IS high) THEN InsulinRequirement IS increase.
19. IF (Intv1GlucoseBegin IS low AND Intv1GlucoseEnd IS medium AND Intv2GlucoseBegin IS low AND Intv2GlucoseEnd IS ex_high) THEN InsulinRequirement IS ex_increase.
20. IF (Intv1GlucoseBegin IS low AND Intv1GlucoseEnd IS medium AND Intv2GlucoseBegin IS medium AND Intv2GlucoseEnd IS high) THEN InsulinRequirement IS increase.
21. IF (Intv1GlucoseBegin IS low AND Intv1GlucoseEnd IS medium AND Intv2GlucoseBegin IS medium AND Intv2GlucoseEnd IS ex_high) THEN InsulinRequirement IS ex_increase.
22. IF (Intv1GlucoseBegin IS low AND Intv1GlucoseEnd IS medium AND Intv2GlucoseBegin IS high AND Intv2GlucoseEnd IS ex_high) THEN InsulinRequirement IS ex_increase.
23. IF (Intv1GlucoseBegin IS low AND Intv1GlucoseEnd IS high AND Intv2GlucoseBegin IS low AND Intv2GlucoseEnd IS medium) THEN InsulinRequirement IS increase.
24. IF (Intv1GlucoseBegin IS low AND Intv1GlucoseEnd IS high AND Intv2GlucoseBegin IS low AND Intv2GlucoseEnd IS high) THEN InsulinRequirement IS increase.
25. IF (Intv1GlucoseBegin IS low AND Intv1GlucoseEnd IS high AND Intv2GlucoseBegin IS low AND Intv2GlucoseEnd IS ex_high) THEN InsulinRequirement IS ex_increase.
26. IF (Intv1GlucoseBegin IS low AND Intv1GlucoseEnd IS high AND Intv2GlucoseBegin IS medium AND Intv2GlucoseEnd IS high) THEN InsulinRequirement IS increase.
27. IF (Intv1GlucoseBegin IS low AND Intv1GlucoseEnd IS high AND Intv2GlucoseBegin IS medium AND Intv2GlucoseEnd IS ex_high) THEN InsulinRequirement IS ex_increase.
28. IF (Intv1GlucoseBegin IS low AND Intv1GlucoseEnd IS high AND Intv2GlucoseBegin IS high AND Intv2GlucoseEnd IS ex_high) THEN InsulinRequirement IS ex_increase.
29. IF (Intv1GlucoseBegin IS low AND Intv1GlucoseEnd IS ex_high AND Intv2GlucoseBegin IS low AND Intv2GlucoseEnd IS medium) THEN InsulinRequirement IS ex_increase.

30. IF (Intv1GlucoseBegin IS low AND Intv1GlucoseEnd IS ex_high AND Intv2GlucoseBegin IS low AND Intv2GlucoseEnd IS high) THEN InsulinRequirement IS ex_increase.
31. IF (Intv1GlucoseBegin IS low AND Intv1GlucoseEnd IS ex_high AND Intv2GlucoseBegin IS low AND Intv2GlucoseEnd IS ex_high) THEN InsulinRequirement IS ex_increase.
32. IF (Intv1GlucoseBegin IS low AND Intv1GlucoseEnd IS ex_high AND Intv2GlucoseBegin IS medium AND Intv2GlucoseEnd IS high) THEN InsulinRequirement IS ex_increase.
33. IF (Intv1GlucoseBegin IS low AND Intv1GlucoseEnd IS ex_high AND Intv2GlucoseBegin IS medium AND Intv2GlucoseEnd IS ex_high) THEN InsulinRequirement IS ex_increase.
34. IF (Intv1GlucoseBegin IS low AND Intv1GlucoseEnd IS ex_high AND Intv2GlucoseBegin IS high AND Intv2GlucoseEnd IS ex_high) THEN InsulinRequirement IS ex_increase.
35. IF (Intv1GlucoseBegin IS medium AND Intv1GlucoseEnd IS high AND Intv2GlucoseBegin IS low AND Intv2GlucoseEnd IS medium) THEN InsulinRequirement IS increase.
36. IF (Intv1GlucoseBegin IS medium AND Intv1GlucoseEnd IS high AND Intv2GlucoseBegin IS low AND Intv2GlucoseEnd IS high) THEN InsulinRequirement IS increase.
37. IF (Intv1GlucoseBegin IS medium AND Intv1GlucoseEnd IS high AND Intv2GlucoseBegin IS low AND Intv2GlucoseEnd IS ex_high) THEN InsulinRequirement IS ex_increase.
38. IF (Intv1GlucoseBegin IS medium AND Intv1GlucoseEnd IS high AND Intv2GlucoseBegin IS medium AND Intv2GlucoseEnd IS high) THEN InsulinRequirement IS increase.
39. IF (Intv1GlucoseBegin IS medium AND Intv1GlucoseEnd IS high AND Intv2GlucoseBegin IS medium AND Intv2GlucoseEnd IS ex_high) THEN InsulinRequirement IS ex_increase.
40. IF (Intv1GlucoseBegin IS medium AND Intv1GlucoseEnd IS high AND Intv2GlucoseBegin IS high AND Intv2GlucoseEnd IS ex_high) THEN InsulinRequirement IS ex_increase.
41. IF (Intv1GlucoseBegin IS medium AND Intv1GlucoseEnd IS ex_high AND Intv2GlucoseBegin IS low AND Intv2GlucoseEnd IS medium) THEN InsulinRequirement IS ex_increase.
42. IF (Intv1GlucoseBegin IS medium AND Intv1GlucoseEnd IS ex_high AND Intv2GlucoseBegin IS low AND Intv2GlucoseEnd IS high) THEN InsulinRequirement IS ex_increase.

43. IF (Intv1GlucoseBegin IS medium AND Intv1GlucoseEnd IS ex_high AND Intv2GlucoseBegin IS low AND Intv2GlucoseEnd IS ex_high) THEN InsulinRequirement IS ex_increase.
44. IF (Intv1GlucoseBegin IS medium AND Intv1GlucoseEnd IS ex_high AND Intv2GlucoseBegin IS medium AND Intv2GlucoseEnd IS high) THEN InsulinRequirement IS ex_increase.
45. IF (Intv1GlucoseBegin IS medium AND Intv1GlucoseEnd IS ex_high AND Intv2GlucoseBegin IS medium AND Intv2GlucoseEnd IS ex_high) THEN InsulinRequirement IS ex_increase.
46. IF (Intv1GlucoseBegin IS medium AND Intv1GlucoseEnd IS ex_high AND Intv2GlucoseBegin IS high AND Intv2GlucoseEnd IS ex_high) THEN InsulinRequirement IS ex_increase.
47. IF (Intv1GlucoseBegin IS high AND Intv1GlucoseEnd IS ex_high AND Intv2GlucoseBegin IS low AND Intv2GlucoseEnd IS medium) THEN InsulinRequirement IS ex_increase.
48. IF (Intv1GlucoseBegin IS high AND Intv1GlucoseEnd IS ex_high AND Intv2GlucoseBegin IS low AND Intv2GlucoseEnd IS high) THEN InsulinRequirement IS ex_increase.
49. IF (Intv1GlucoseBegin IS high AND Intv1GlucoseEnd IS ex_high AND Intv2GlucoseBegin IS low AND Intv2GlucoseEnd IS ex_high) THEN InsulinRequirement IS ex_increase.
50. IF (Intv1GlucoseBegin IS high AND Intv1GlucoseEnd IS ex_high AND Intv2GlucoseBegin IS medium AND Intv2GlucoseEnd IS high) THEN InsulinRequirement IS ex_increase.
51. IF (Intv1GlucoseBegin IS high AND Intv1GlucoseEnd IS ex_high AND Intv2GlucoseBegin IS medium AND Intv2GlucoseEnd IS ex_high) THEN InsulinRequirement IS ex_increase.
52. IF (Intv1GlucoseBegin IS high AND Intv1GlucoseEnd IS ex_high AND Intv2GlucoseBegin IS high AND Intv2GlucoseEnd IS ex_high) THEN InsulinRequirement IS ex_increase.
53. IF (Intv1GlucoseBegin IS ex_high AND Intv1GlucoseEnd IS high AND Intv2GlucoseBegin IS ex_high AND Intv2GlucoseEnd IS high) THEN InsulinRequirement IS decrease.
54. IF (Intv1GlucoseBegin IS ex_high AND Intv1GlucoseEnd IS high AND Intv2GlucoseBegin IS ex_high AND Intv2GlucoseEnd IS medium) THEN InsulinRequirement IS decrease.
55. IF (Intv1GlucoseBegin IS ex_high AND Intv1GlucoseEnd IS high AND Intv2GlucoseBegin IS ex_high AND Intv2GlucoseEnd IS low) THEN InsulinRequirement IS ex_decrease.

56. IF (Intv1GlucoseBegin IS ex_high AND Intv1GlucoseEnd IS high AND Intv2GlucoseBegin IS high AND Intv2GlucoseEnd IS medium) THEN InsulinRequirement IS decrease.
57. IF (Intv1GlucoseBegin IS ex_high AND Intv1GlucoseEnd IS high AND Intv2GlucoseBegin IS high AND Intv2GlucoseEnd IS low) THEN InsulinRequirement IS ex_decrease.
58. IF (Intv1GlucoseBegin IS ex_high AND Intv1GlucoseEnd IS high AND Intv2GlucoseBegin IS medium AND Intv2GlucoseEnd IS low) THEN InsulinRequirement IS decrease.
59. IF (Intv1GlucoseBegin IS ex_high AND Intv1GlucoseEnd IS medium AND Intv2GlucoseBegin IS ex_high AND Intv2GlucoseEnd IS high) THEN InsulinRequirement IS decrease.
60. IF (Intv1GlucoseBegin IS ex_high AND Intv1GlucoseEnd IS medium AND Intv2GlucoseBegin IS ex_high AND Intv2GlucoseEnd IS medium) THEN InsulinRequirement IS decrease.
61. IF (Intv1GlucoseBegin IS ex_high AND Intv1GlucoseEnd IS medium AND Intv2GlucoseBegin IS ex_high AND Intv2GlucoseEnd IS low) THEN InsulinRequirement IS ex_decrease.
62. IF (Intv1GlucoseBegin IS ex_high AND Intv1GlucoseEnd IS medium AND Intv2GlucoseBegin IS high AND Intv2GlucoseEnd IS medium) THEN InsulinRequirement IS decrease.
63. IF (Intv1GlucoseBegin IS ex_high AND Intv1GlucoseEnd IS medium AND Intv2GlucoseBegin IS high AND Intv2GlucoseEnd IS low) THEN InsulinRequirement IS ex_decrease.
64. IF (Intv1GlucoseBegin IS ex_high AND Intv1GlucoseEnd IS medium AND Intv2GlucoseBegin IS medium AND Intv2GlucoseEnd IS low) THEN InsulinRequirement IS decrease.
65. IF (Intv1GlucoseBegin IS ex_high AND Intv1GlucoseEnd IS low AND Intv2GlucoseBegin IS ex_high AND Intv2GlucoseEnd IS high) THEN InsulinRequirement IS ex_decrease.
66. IF (Intv1GlucoseBegin IS ex_high AND Intv1GlucoseEnd IS low AND Intv2GlucoseBegin IS ex_high AND Intv2GlucoseEnd IS medium) THEN InsulinRequirement IS ex_decrease.
67. IF (Intv1GlucoseBegin IS ex_high AND Intv1GlucoseEnd IS low AND Intv2GlucoseBegin IS ex_high AND Intv2GlucoseEnd IS low) THEN InsulinRequirement IS ex_decrease.
68. IF (Intv1GlucoseBegin IS ex_high AND Intv1GlucoseEnd IS low AND Intv2GlucoseBegin IS high AND Intv2GlucoseEnd IS medium) THEN InsulinRequirement IS ex_decrease.

69. IF (Intv1GlucoseBegin IS ex_high AND Intv1GlucoseEnd IS low AND Intv2GlucoseBegin IS high AND Intv2GlucoseEnd IS low) THEN InsulinRequirement IS ex_decrease.
70. IF (Intv1GlucoseBegin IS ex_high AND Intv1GlucoseEnd IS low AND Intv2GlucoseBegin IS medium AND Intv2GlucoseEnd IS low) THEN InsulinRequirement IS ex_decrease.
71. IF (Intv1GlucoseBegin IS high AND Intv1GlucoseEnd IS medium AND Intv2GlucoseBegin IS ex_high AND Intv2GlucoseEnd IS high) THEN InsulinRequirement IS decrease.
72. IF (Intv1GlucoseBegin IS high AND Intv1GlucoseEnd IS medium AND Intv2GlucoseBegin IS ex_high AND Intv2GlucoseEnd IS medium) THEN InsulinRequirement IS decrease.
73. IF (Intv1GlucoseBegin IS high AND Intv1GlucoseEnd IS medium AND Intv2GlucoseBegin IS ex_high AND Intv2GlucoseEnd IS low) THEN InsulinRequirement IS ex_decrease.
74. IF (Intv1GlucoseBegin IS high AND Intv1GlucoseEnd IS medium AND Intv2GlucoseBegin IS high AND Intv2GlucoseEnd IS medium) THEN InsulinRequirement IS decrease.
75. IF (Intv1GlucoseBegin IS high AND Intv1GlucoseEnd IS medium AND Intv2GlucoseBegin IS high AND Intv2GlucoseEnd IS low) THEN InsulinRequirement IS ex_decrease.
76. IF (Intv1GlucoseBegin IS high AND Intv1GlucoseEnd IS medium AND Intv2GlucoseBegin IS medium AND Intv2GlucoseEnd IS low) THEN InsulinRequirement IS decrease.
77. IF (Intv1GlucoseBegin IS high AND Intv1GlucoseEnd IS low AND Intv2GlucoseBegin IS ex_high AND Intv2GlucoseEnd IS high) THEN InsulinRequirement IS ex_decrease.
78. IF (Intv1GlucoseBegin IS high AND Intv1GlucoseEnd IS low AND Intv2GlucoseBegin IS ex_high AND Intv2GlucoseEnd IS medium) THEN InsulinRequirement IS ex_decrease.
79. IF (Intv1GlucoseBegin IS high AND Intv1GlucoseEnd IS low AND Intv2GlucoseBegin IS ex_high AND Intv2GlucoseEnd IS low) THEN InsulinRequirement IS ex_decrease.
80. IF (Intv1GlucoseBegin IS high AND Intv1GlucoseEnd IS low AND Intv2GlucoseBegin IS high AND Intv2GlucoseEnd IS medium) THEN InsulinRequirement IS ex_decrease.
81. IF (Intv1GlucoseBegin IS high AND Intv1GlucoseEnd IS low AND Intv2GlucoseBegin IS high AND Intv2GlucoseEnd IS low) THEN InsulinRequirement IS ex_decrease.
82. IF (Intv1GlucoseBegin IS high AND Intv1GlucoseEnd IS low AND Intv2GlucoseBegin IS medium AND Intv2GlucoseEnd IS low) THEN InsulinRequirement IS ex_decrease.
83. IF (Intv1GlucoseBegin IS medium AND Intv1GlucoseEnd IS low AND Intv2GlucoseBegin IS ex_high AND Intv2GlucoseEnd IS high) THEN InsulinRequirement IS decrease.

84. IF (Intv1GlucoseBegin IS medium AND Intv1GlucoseEnd IS low AND Intv2GlucoseBegin IS ex_high AND Intv2GlucoseEnd IS medium) THEN InsulinRequirement IS decrease.

85. IF (Intv1GlucoseBegin IS medium AND Intv1GlucoseEnd IS low AND Intv2GlucoseBegin IS ex_high AND Intv2GlucoseEnd IS low) THEN InsulinRequirement IS ex_decrease.

86. IF (Intv1GlucoseBegin IS medium AND Intv1GlucoseEnd IS low AND Intv2GlucoseBegin IS high AND Intv2GlucoseEnd IS medium) THEN InsulinRequirement IS decrease.

87. IF (Intv1GlucoseBegin IS medium AND Intv1GlucoseEnd IS low AND Intv2GlucoseBegin IS high AND Intv2GlucoseEnd IS low) THEN InsulinRequirement IS ex_decrease.

88. IF (Intv1GlucoseBegin IS medium AND Intv1GlucoseEnd IS low AND Intv2GlucoseBegin IS medium AND Intv2GlucoseEnd IS low) THEN InsulinRequirement IS decrease.
